# Cu─X Bonds Regulated Conduction and Polarization Loss in Conductive Metal‐Organic Framework Under Electromagnetic Field

**DOI:** 10.1002/advs.202508379

**Published:** 2025-06-10

**Authors:** Siyao Cheng, Qinglin Zhou, Daohu Sheng, Wei Dong, Jinhu Dou, Yuanbiao Huang, Rong Cao, Aming Xie, Roland A. Fischer, Soumya Mukherjee, Weijin Li

**Affiliations:** ^1^ MIIT Key Laboratory of Advanced Display Materials and Devices & Materials Physical and Chemical Research and Practice Center College of Materials Science and Engineering Nanjing University of Science and Technology Nanjing 210094 P. R. China; ^2^ State Key Laboratory of Structural Chemistry Fujian Institute of Research on the Structure Chinese Academy of Sciences Fuzhou 350002 P. R. China; ^3^ School of Chemistry and Chemical Engineering Nanjing University of Science and Technology Nanjing 210094 P. R. China; ^4^ School of Safety Science and Engineering Nanjing University of Science and Technology Nanjing 210094 P. R. China; ^5^ Bernal Institute and Department of Chemical Sciences University of Limerick Limerick V94 T9PX Ireland; ^6^ School of Materials Science and Engineering Peking University Zonghe Science Building Nanjing 700871 P. R. China; ^7^ Chair of Inorganic and Metal‐organic Chemistry Department of Chemistry & School of Natural Sciences Technical University of Munich Lichtenbergstrasss 4 85748 Garching Germany

**Keywords:** conduction loss, conductive metal–organic frameworks, electromagnetic wave absorption, inter‐layer charge transport, intra‐layer charge transport, polarization loss

## Abstract

Conduction and polarization are known to profoundly impact conductive metal–organic frameworks (c‐MOFs) for their applications in electromagnetic wave (EMW) absorption. Albeit a few advances along c‐MOF platforms in enhancing their EMW absorption performances, reticular modulation‐led inter/intra‐layer conduction and polarization loss remains an unmet challenge. To address this, a ligand substitution‐guided bottom‐up structural control strategy is introduced to study the depth of reticular modulation‐led inter/intra‐layer conduction and polarization loss in c‐MOFs under an electromagnetic (EM) field. A family of triphenylene‐X ligands (X = −NH_2_, −OH, and −SH) is harnessed to afford an isoreticular family of three Cu‐based c‐MOFs. Thanks to the distinct Cu─X bonds, such a platform allowed to systematically study the synergistic features of conduction and polarization loss in EMW absorption enhancement. One of the trio, Cu_3_(HITP)_2_ (X = −NH_2_; HITP, 2,3,6,7,10,11‐hexahydroxytriphenylene) is identified with an optimal EM loss capacity under the EM field, achieving a record‐high reflection loss of −63.03 dB in the effective absorption range of 3–18 GHz band. Setting up a new benchmark for EM loss among c‐MOFs, this study introduces a way to leverage control in the charge mobility characteristics of Cu─X bonds relative to the dielectric losses at both molecular and atomic scales.

## Graphical abstract

A ligand regulation strategy by modifying functional motifs (X = −NH_2_, −OH, and −SH) in triphenylene is pioneered and developed to afford Cu─X bonds, achieving a synergistic conduction and polarisation loss mechanism under an electromagnetic field for enhanced electromagnetic wave absorption.

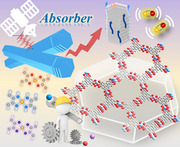

## 1 Introduction

A fast‐emerging class of metal‐organic frameworks (MOFs), conductive metal‐organic frameworks (c‐MOFs) feature the “*best of both Worlds*”, high charge transport and crystalline porous architectures, thereby allowing bottom‐up control to chemists and crystal engineers.^[^ ^1^, ^2^ ^]^ Thanks to their periodic porosity (often resulting in high surface areas), tunable high charge mobility and/or electrical conductivity, c‐MOFs are poised to be the energy‐efficient alternatives to the state‐of‐the‐art in chemo‐resistive sensing, supercapacitors, electrocatalysis, and electronic devices, among others.^[^ ^3^, ^4^ ^]^ Considering their potential to lower the consumption of excess electromagnetic wave (EMW) stemming from the modern 5G telecommunication technologies,^[^ ^5^, ^6^ ^]^ the prominence of c‐MOFs has further amplified of late.^[^ ^7^, ^8^ ^]^ EMW consumption, or EMW absorption, is known to arise from conduction loss‐dipolar/molecular/atomic/electronic/defect‐induced/ionic polarization, one or more of the various origins thereof.^[^ ^9^ ^]^ Interestingly, the tunable bandgap and high charge mobility/electrical conductivity of the c‐MOFs enable them to realize EMW absorption through conduction‐dominated EMW loss. In this context, advances in controlling the electronic conduction of c‐MOFs have recently come to the fore, where regulating the metal center and lattice interlayer ratios emerged as crucial in efficiently absorbing EMW.^[^ ^8^, ^10^ ^]^ However, tuning the dipolar/molecular polarization in c‐MOFs is an underexplored approach with regard to understanding their structure‐polarization‐EMW absorption relationships.^[^ ^11^ ^]^

Typically built from metal ions (or clusters) and conjugated organic ligands, c‐MOFs are crystalline porous frameworks connected by metal‐ligand coordination bonds. When designed right, c‐MOFs exhibit properties that benefit from either their inorganic or organic moieties. The inorganic‐organic duality and long‐range order of c‐MOFs offer a unique scope to reticular chemists of fine‐tuning their polarizability. Tweaking the metal ions along a few prototypical c‐MOF platforms have resulted in incremental conduction and polarization loss under EM field.^[^ ^12^, ^13^ ^]^In this context, a strategy that can enable chemists to regulate the EMW‐induced inter‐/intra‐layer charge transport, *vis‐à‐vis* to thereby elicit an optimal conduction and polarization loss presents the next frontier.^[^ ^14^ ^]^

Herein, we report on a *prêt‐à‐porter* ligand engineering strategy to obtain a family of three c‐MOFs. Stitched together from periodic Cu‐X_4_ (X = N (amino), O (hydroxyl), S (thiol)) coordinating scaffolds that are comparable, three along this isoreticular platform are probed with regard to their EMW absorption loss mechanisms and performances thereof.^[^ ^15^ ^]^ These 2D(2D) layered c‐MOFs feature an identical honeycomb (hcb) topology (point group 6^3^), featuring 18π electron‐delocalized triphenylene blocks with terminal functional groups X.^[^ ^16^ ^]^ Each of the X groups, N, O and S is found to further coordinate to Cu^(II)^ to afford square planar structural building units. Unison of these structural aspects, simply put, renders an intrinsic amenability to Cu─X bond tuning‐derived property changes along the isoreticular c‐MOF platform of three.^[^ ^17^, ^18^ ^]^ Each structure consists of a layered hcb network structure with in‐plane extended π‐conjugation and out‐of‐plane π‐orbital overlap. This twofold directional nature is primed to allow efficient charge transport along both directions. That −NH_2_, −OH and −SH present at the organic ligands termini is likely to influence the d−π conjugated system upon coordination to Cu^(II)^, inter− and intra−layer charge transport capacity of c‐MOFs under EM field will also be contingent on these points of influence. Unlike the studies of functional group introduction and heteroatom doping, this work focuses on probing the strategies for the regulation of Cu—X coordination bonds. The role of different coordination atoms simultaneously affects the electronic structure of the central metal as well as the long‐range in‐plane‐out‐of‐plane charge distribution at the molecular level. Delving into the interlayer and intralayer charge transport characteristics that is likely an outcome of the distinct electric charge distribution, impacts on the three c‐MOFs’ dielectric properties and EMW absorption performances were systematically investigated.

## 2 Results and Discussion

### 2.1 Structural and Coordination Environment of the Cu─X Regulated c‐MOFs

**Figure 1:**
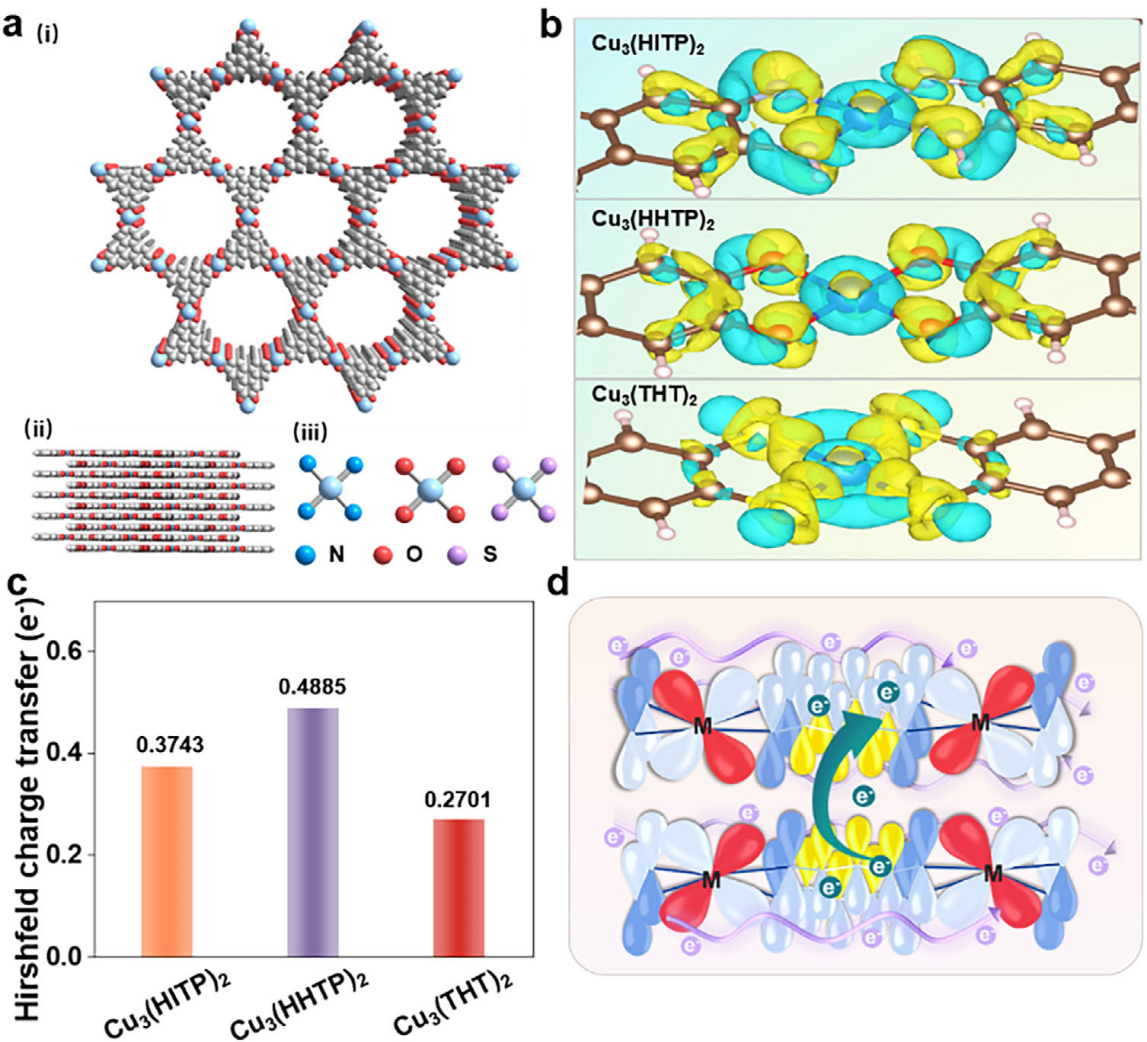
Phase structure and electron distribution. a) The hexagonal crystal structure of Cu_3_(HITP)_2_, Cu_3_(HHTP)_2_, and Cu_3_(THT)_2_ (i), tiered stacking pattern (ii) and schematic diagram of Cu‐X_4_ coordination unit (iii). b) Charge density distribution differences between the three c‐MOFs, along their three distinct ligands (HITP, HHTP, THT), and Cu. c) Hirshfeld charge transfer in Cu_3_(HITP)_2_, Cu_3_(HHTP)_2_, and Cu_3_(THT)_2_ (determined for Cu). d) Schematic representation of two charge transport pathways.

**Figure 2:**
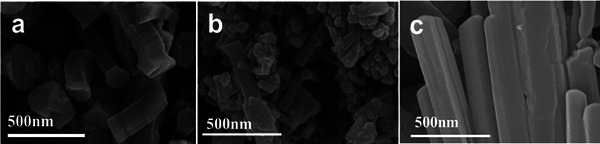
The SEM micrographs for a) Cu_3_(HHTP)_2_, b) Cu_3_(HITP)_2_ and c) Cu_3_(THT)_2_.

Three 2D c‐MOFs, Cu_3_(HITP)_2_, Cu_3_(HHTP)_2_, and Cu_3_(THT)_2_, (HITP, 2,3,6,7,10,11‐hexahydroxytriphenylene; HHTP, 2,3,6,7,10,11‐hexahydroxytriphenylene; THT, 2,3,6,7,10,11‐hexathiophenylene) are prepared through one‐pot syntheses, following literature protocols (**Figure** **1**a, details in the Supporting Information, sections 1.2−1.4).^[^ ^19^, ^20^, ^21^ ^]^ As mentioned, the typical isoreticular hcb topology c‐MOFs, Cu_3_(HITP)_2_, Cu_3_(HHTP)_2_, and Cu_3_(THT)_2_ exhibit distinct coordination moieties, viz., Cu─N, Cu─O and Cu─S respectively (Figure S1a, Supporting Information). Scanning electron microscopy (SEM) micrographs reveal that all three MOFs have nano‐hexagonal prism as the uniform polycrystalline morphology and their lengths are at the micrometer level (**Figure** **2**; Figure S2, Supporting Information). The clear lattice stripes taken under high‐resolution transmission electron microscopy (HR‐TEM) images indicate that a high degree of extended order in each c‐MOF (Figures S1–S4, Supporting Information). The lattice distances of 3.2 and 3.5 Å noted in Cu_3_(HITP)_2_ and Cu_3_(THT)_2_ can be indexed to the interlayer distances of their (201) and (001) crystal planes, respectively (Figure S1c,g, Supporting Information). The crystal structure was also demonstrated by powder X‐ray diffraction (PXRD) patterns (Figure S5, Supporting Information). Cryogenic N_2_ at 77 K adsorption isotherm‐based Brunauer‐Emmett‐Teller (BET) specific surface areas revealed the increasing trend of Cu_3_(THT)_2_: 31.76 << Cu_3_(HHTP)_2_: 206.54 < Cu_3_(HITP)_2_: 232.78 (areas expressed in m^2^/g), further indicating the exclusive nature of staggered interlayer stacking in Cu_3_(THT)_2_ (Figure S6, Supporting Information).^[^ ^22^ ^]^ This cross‐stacking is likely to be an outcome of the sulfur atom's largest atomic radius versus the radii of N and O (atomic radii of X (R_X_): R_S_ = 1.06 Å; R_N_ = 0.7 Å; R_O_ = 0.66 Å).^[^ ^23^ ^]^ The staggered phase has been identified with a lower conductivity than the eclipsed phases, notwithstanding with their crystallographic orientations.^[^ ^18^ ^]^ As a consequence, the staggered stacking pattern is likely to reduce the interlayer charge transfer, including the current 2D c‐MOF congeners.^[^ ^24^ ^]^

The Cu─X bonds and charge distribution of c‐MOFs were studied by Fourier transform infrared spectroscopy (FT‐IR) and X‐ray photoelectron spectroscopy (XPS). The relative weakness of C—NH_2_, C—OH, and C—SH FT‐IR peak intensities for Cu─X in the c‐MOFs versus those in the pristine ligands suggests Cu^(II)^ coordination to the π‐conjugated triphenylene‐based ligands that enables extended electron delocalization across the c‐MOF structures (Figure S7, Supporting Information). Such electron delocalization is known to benefit intralayer charge transport.^[^ ^25^ ^]^Compared to the Cu *2p_1/2_* ^(II)^ peak in Cu_3_(HHTP)_2_ at 953.1 eV, the Cu *2p_1/2_* ^(II)^ peaks in Cu_3_(HITP)_2_ and Cu_3_(THT)_2_ were found to be shifted by 0.2 and 0.4 eV toward lower binding energies, respectively (Figures S8 and S9, Supporting Information). This peak shift indicates that the degree of electron transfer from Cu^(II)^ to O atoms is stronger relative to the S and N atoms, likely related to the three heteroatoms’ different electronegativities.^[^ ^26^ ^]^ Stronger electronegativity of X (such as, in O) leads to pronounced concentration of electrons on it, resulting in a higher Cu─X charge separation, optimizing the dipoles thereof. On the flipside, localized concentration of electrons culminates in an uneven charge distribution in the c‐MOF plane, thus contributing negatively to charge transport.

Coordination environments of the prepared c‐MOFs were further investigated using X‐ray absorption fine structure spectroscopy (EXAFS). The normalized X‐ray absorption near‐edge fine structure (XANES) spectra of Cu_3_(HITP)_2_ at the Cu K‐edge reveal a shoulder peak at 8982 eV, which belongs to the characteristic peak of Cu^(I)^ (Figure S10, Supporting Information).^[^ ^27^, ^28^ ^]^ The R‐space fitting curves were found to be in agreement with the experimental spectra. As shown in the Table S2 and Figure S11 (Supporting Information), coordination numbers for each of the Cu─X moieties was determined to be 4, confirming the presence of periodic Cu‐X_4_ structures (Figure 1a).^[^ ^29^ ^]^

### 2.2 Electronic and Charge Transport Behavior of the Cu─X Regulated c‐MOFs

Electron density distribution maps and Hirshfeld charge transfer diagrams were employed to critically interrogate the electronic and structural properties of the c‐MOFs.^[^ ^30^ ^]^ Given the direct bonding of ligand atoms to the metal centers, systematic tripartite variation in the ligand atoms was found to significantly influence the electron density of the metal centers. As shown in Figure 1b, the metal sites exhibited charge depletion, whereas electronegativity for each of the ligating atoms (X) was evident. Electron density near the metal ions was also found to be localized. The electron density center on the coordinating atom indicates the transfer of electrons to the carbon atom, which is consistent with the electron transfer charge accumulation. In Cu_3_(HITP)_2_, electron density was found to be concentrated around the N atoms, indicating strong charge transfer facilitated by the d−π conjugation effect.^[^ ^31^ ^]^ In Cu_3_(HHTP)_2_, electron density around the O atoms was found to be more concentrated, whereas electron density near the central metal ions was found higher than the N‐containing ligand HITP, indicating a stronger electron‐attracting ability of the oxygen atoms. However, in Cu_3_(THT)_2_, electron density around the S atoms was found to be the most dispersed in nature, whereas the electron density near the central metal ions was found to hit the lowest. This observation was consistent with S atoms featuring the minimum electronegativity, among the three X variants. The ligand's ability to attract electrons significantly affects the charge distribution within the in‐plane of 2D c‐MOFs. The Hirshfeld charge transfer diagrams illustrate electron transfer among the three c‐MOFs (Figure 1c). from the central Cu6, O atoms (O32, O47, O62, O16) receive *ca*. 0.4 e^−^, N atoms (N16, N62, N32, N47) receive *ca*. 0.3 e^−^, and S atoms (S16, S62, S32, S47) receive ≈ 0.2 e^−^ (Figure S12, Supporting Information). Charge densities of the Cu centers were found to follow the increasing trend, Cu_3_(HHTP)_2_ < Cu_3_(HITP)_2_ < Cu_3_(THT)_2_, in line with those of the XPS results, and thus exemplifying an in‐plane charge distribution across the studied c‐MOFs. O with the highest electronegativity attracts the most electrons, that is, the electron cloud significantly surrounds the Cu─O bonds. On the contrary, S with the lowest electronegativity draws the fewest electrons in, that is, the electron cloud does not accumulate around the Cu─S bonds.^[^ ^32^ ^]^ Here the dielectric properties could be optimized due to the inhomogeneous charge distribution that in turn, resulted in the distribution of intramolecular local dipole moments.

**Figure 3:**
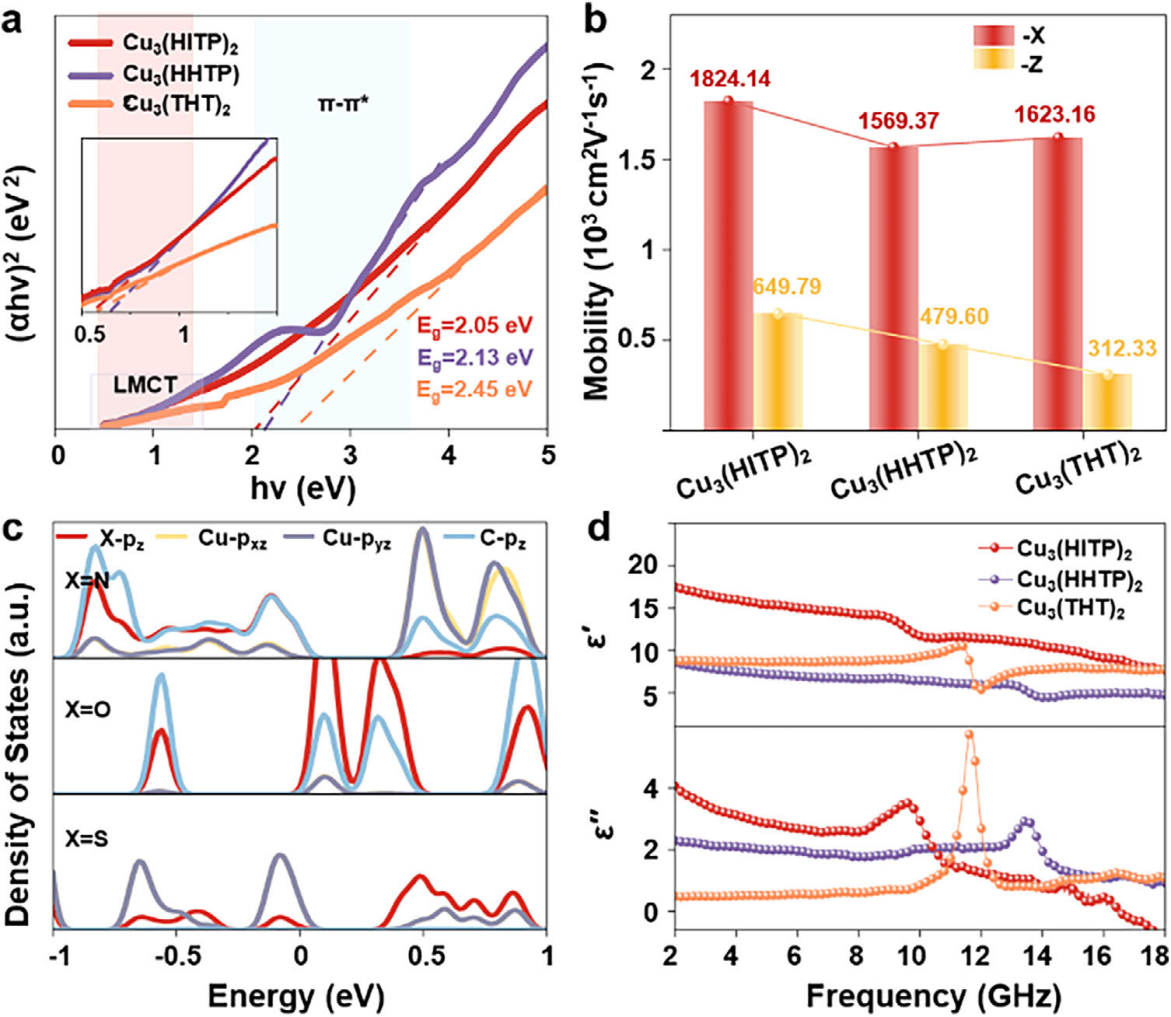
Charge transport and dielectric properties. a) Tauc fits presenting the optical bandgaps in Cu_3_(HITP)_2_, Cu_3_(HHTP)_2_, and Cu_3_(THT)_2_ (the inset zoomed view shows the bandgap (E_g_) located in the LMCT section). b) The Carrier mobility calculation of the Cu_3_(HITP)_2_, Cu_3_(HHTP)_2_, and Cu_3_(THT)_2_ (x‐direction and z‐direction). c) The simulated density of states for Cu_3_(HITP)_2_, Cu_3_(HHTP)_2_, and Cu_3_(THT)_2_. d) The real part (ε′) and imaginary part (ε′′) of permittivity.

From the differential charge density diagrams and Hirshfeld charge transfer profiles, Cu_3_(HITP)_2_ was found to exhibit a strong charge delocalization and more electron transfer to the conjugated C, factors closely related to the d−π conjugation effect (occurring between the ligand π‐electrons and the metal d‐electrons) (Figure 1b; Figure S12, Supporting Information). Using the four‐probe method, the electrical conductivities of Cu_3_(HITP)_2_, Cu_3_(HHTP)_2_, and Cu_3_(THT)_2_ were determined to be 2.03 ± 0.4 S m^−1^, 0.47 ± 0.12 S m^−1^, and 0.075 ± 0.007 S m^−1^, respectively (Figures S14 and S15, Supporting Information). As shown in Figure 1d, two distinct transport pathways are identified in the c‐MOFs: inter‐layer and intra‐layer charge transport. It can be deduced that the electrical conductivity in 2D c‐MOFs is mainly governed by their inter‐ and intra‐layer charge transport properties.^[^ ^2^ ^]^ Electronic states in these c‐MOFs were further elucidated using diffuse reflectance ultraviolet‐visible spectroscopy (DR UV‐Vis). Fitting the Tauc plots revealed two distinct energy states: one corresponding to the ligand‐to‐metal intralayer charge transport (LMCT), and the other to π‐π* interlayer charge transfer occurring between the organic ligands (**Figure** **3**a).^[^ ^33^ ^]^ In literature, interlayer charge transfer is often controlled by supramolecular stacking of the 2D layers.^[^ ^34^ ^]^ Indeed, commensurate stacking of layers allows for the formation of 1D charge transfer channels.^[^ ^34^ ^]^ Cu_3_(THT)_2_, which exhibits a staggered interlayer stacking as described above, demonstrates a high bandgap of 2.45 eV in the π‐π* region, indicating that interlayer charge transport is impeded by the staggered stacking pattern and large interlayer distances (3.5 Å).^[^ ^35^, ^36^, ^37^ ^]^ In contrast, the slip‐parallel stacked Cu_3_(HITP)_2_ and Cu_3_(HHTP)_2_ exhibit similar layer spacings of 3.2 Å and interlayer electron gaps of 2.13 and 2.05 eV, respectively. This implies that the efficient stacking approach enhances the interlayer charge transport capacity. In addition, the more rigid ligating atoms (N, and O) facilitate to retain the planarity, thereby allowing extended π‐conjugation.^[^ ^38^, ^39^ ^]^ The narrower bandgap of N originates from the enhanced π ‐ π * interactions, likely stemming from the Cu─X bonds drawing in stronger delocalization of electrons to the conjugated triphenylene blocks. Carrier mobility calculations confirm on a theoretical level that Cu_3_(HITP)_2_ has the fastest carrier mobility in the z‐direction (649.79 cm^2^V^−1^s^−1^) (i.e., out‐of‐plane direction), while Cu_3_(THT)_2_ exhibits the worst carrier mobility (312.33 cm^2^V^−1^s^−1^). It is further evidenced that the interlayer charge transport path of Cu_3_(THT)_2_ is blocked (Figure 3b).

Meanwhile, the intralayer charge transport pathway is mainly through‐bond via metal‐to‐ligand/ligand‐to‐metal charge transfer pathway. In principle, the through‐bond transport pathway relies upon the energy overlap between the covalent bonding orbitals of the metal and the ligand, with a larger d−π electron cloud density cross‐coverage implying stronger conjugation, that in turn, translates into faster electron transfer.^[^ ^40^ ^]^ The intralayer d−π charge transport, as represented in Tauc coordinates, reveals optical bandgaps of 0.54 eV for Cu_3_(HITP)_2_, 0.64 eV for Cu_3_(HHTP)_2_, and 0.57 eV for Cu_3_(THT)_2_. The optical bandgaps derived trend matches well with the Density Functional Theory (DFT)‐calculated in‐plane electron gaps of 0.39, 0.62, and 0.40 eV for Cu₃(HITP)₂, Cu₃(HHTP)₂, and Cu₃(THT)₂, respectively (Figure S16¸Supporting Information). The electronic energy band structures support that both Cu₃(HITP)₂ and Cu₃(THT)₂ exhibit pronounced electronic band dispersion.^[^ ^41^, ^42^ ^]^ The N and S atoms in Cu_3_(HITP)_2_ and Cu_3_(THT)_2_ exhibit a strong d−π conjugation with Cu, and further enhance their Intra‐plane carrier mobility (Figure 3b). Improvement in their carrier mobility is attributed to the optimal energy alignment between the amino (or thiol) groups and the metal orbitals, coupled with increased orbital overlap.^[^ ^31^ ^]^ For Cu₃(HITP)₂, the density of states near the Fermi energy level is predominantly derived from the delocalized p_z_ orbitals of carbon, the d_xz_/d_yz_ orbitals of copper, and the p_z_ orbitals of nitrogen (Figure 3c). Overlap between these orbitals represents the presence of d− π conjugation.^[^ ^43^, ^44^ ^]^A stronger coupling between the d_xz_/d_yz_ orbitals of Cu and the p_z_ orbitals of N (in HITP) results in higher energy levels for the Cu d_xz_/d_yz_ orbitals, rendering the electronic states more delocalized. The observed strong d−π conjugation is indeed corroborated well by the fact that Cu₃(HITP)₂ exhibits the most pronounced absorption in the near‐IR region (Figure S17, Supporting Information).^[^ ^45^ ^]^

Given that Cu^2^⁺ is the only source of Cu in the system, the Cu⁺ generated through the ligand‐initiated redox process reflects the interactions between the metal center and the ligand.^[^ ^46^, ^47^ ^]^ A lower Cu^2^⁺/Cu⁺ ratio could result in a stronger electronic coupling between the copper ion and the ligand, causing more electrons to be transferred from copper to the ligand. Cu₃(HITP)₂ notches the smallest Cu^2^⁺/Cu⁺ ratio as demonstrated above from both XPS and XAFS (Table S1; Figures S9 and S10, Supporting Information). This ratio correlates with both the in‐plane bandgap calculated by DFT and the optical bandgap calculated by UV‐Vis spectral signatures. This finding was also supported from electron paramagnetic resonance (EPR) spectroscopy experiments (Figure S18, Supporting Information). The ultrafine structures for the c‐MOFs were analyzed at cryogenic temperatures using liquid nitrogen, revealing broad peaks with g‐factors ranging between 2.28 and 2.04. This was found to be the characteristic of Cu^2^⁺ in a square planar coordination environment.^[^ ^48^ ^]^ Cu₃(HHTP)₂ exhibits the strongest EPR signal, corresponding to a higher concentration of Cu^2^⁺, which is also why Cu₃(HITP)₂ registers the weakest signal. These distinct spectral features influence the coordination environment that arise from the metal ion's electronic structure. Moreover, the peak at g = 2.004 in the EPR spectrum confirms the presence of organic π radicals generated from oxidation of the HHTP, HITP, and THT ligands.^[^ ^49^, ^50^, ^51^ ^]^ The ligand groups (—OH, —NH₂, —SH) influence the ligand oxidation states. A higher oxidation state results in a greater number of free carriers, thereby enhancing conductivity.^[^ ^52^, ^53^ ^]^

### 2.3 Electromagnetic Wave Absorption Performance of the Cu─X Regulated c‐MOFs

To elucidate the electromagnetic wave absorption characteristics of the obtained c‐MOFs, their complex permittivity (ε_r_ = ε′ – jε′′) and complex permeability (µ_r_ = µ′ – jµ′′) were investigated. The real part (ε′ and µ′) reflects the material's ability to store electromagnetic energy, while the imaginary part (ε′′ and µ′′) represents its energy dissipation capacity. The complex dielectric permittivity serves as a key parameter for evaluating the material's dielectric properties. The values of ε′ for Cu_3_(HITP)_2_, Cu_3_(HHTP)_2_ and Cu_3_(THT)_2_ range from 17.50 to 7.67, 8.54 to 4.39, and 8.77 to 7.73 at the frequency of 2 to 18 GHz, respectively. Notably, the ε′′ value was found to rise with increasing conductivity, following the decreasing trend Cu_3_(HITP)_2_ > Cu_3_(HHTP)_2_ > Cu_3_(THT)_2_ (Figure 3d). Each of the c‐MOFs presents a pronounced polarization relaxation peak in both the ε′′ and the dielectric loss factor (tanδ_ε_) versus frequency curves, indicating the presence of polarization loss (Figure S20, Supporting Information).

**Figure 4:**
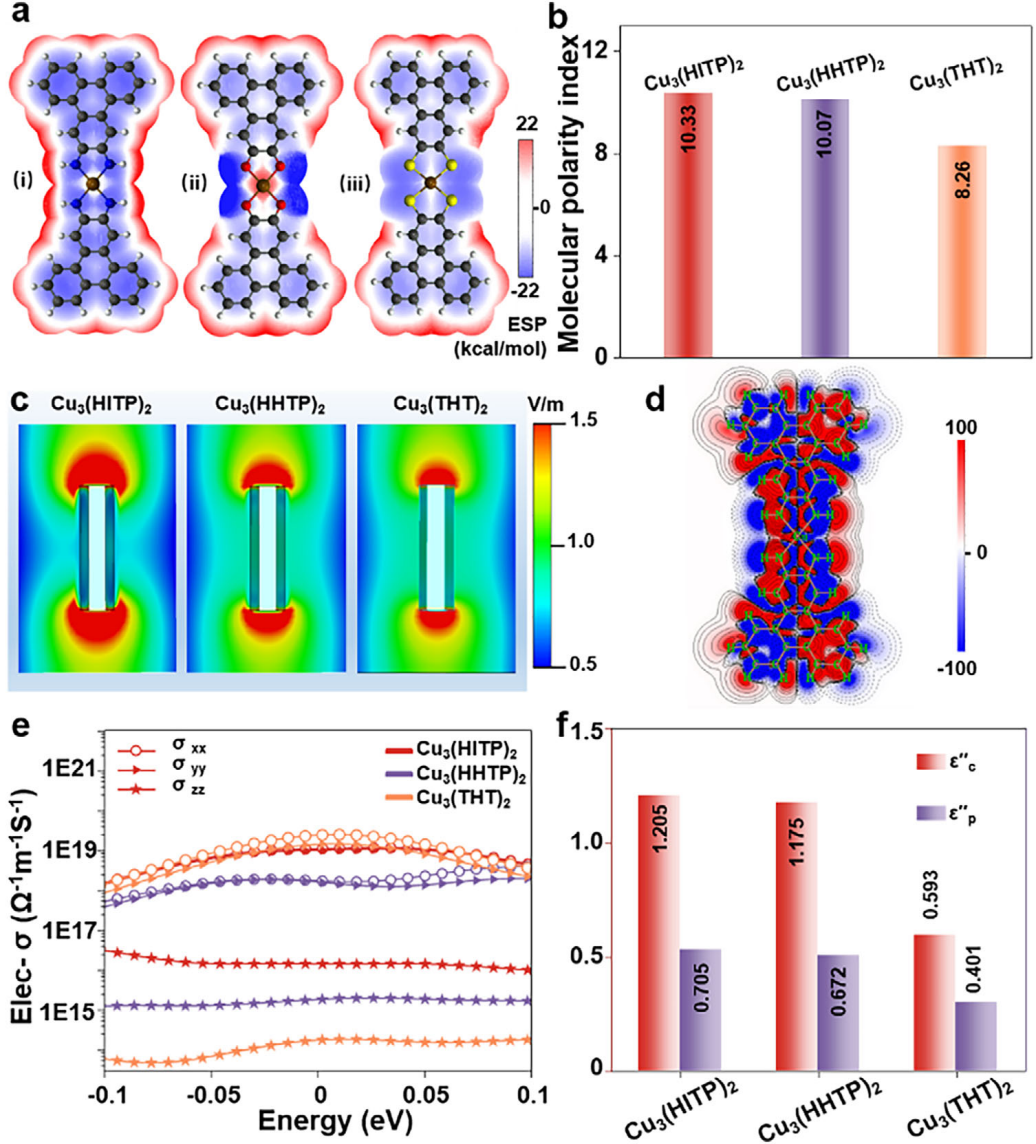
Polarization features. a,b) The electrostatic potential maps (a) and the molecular polarity index (b) Obtained by DFT simulation of Cu_3_(HITP)_2_ (i), Cu_3_(HHTP)_2_ (ii), and Cu_3_(THT)_2_ (iii). c) Simulated electric field distribution maps of Cu_3_(HITP)_2_, Cu_3_(HHTP)_2_, and Cu_3_(THT)_2_. d) Hyperpolarizability density contour plot of the Cu_3_(HITP)_2_. e) Boltzmann transport theory calculations of electronic conductivity (average values in the *xx, yy* and *zz* directions) of the Cu_3_(HITP)_2_, Cu_3_(HHTP)_2_, and Cu_3_(THT)_2_. f) Determination of ε_c_″ and ε_p_″ in Cu_3_(HITP)_2_, Cu_3_(HHTP)_2_, and Cu_3_(THT)_2_.

The electrostatic potential (ESP) map of a single repeating unit indicates that the distribution of electron‐deficient regions is concentrated at the non‐conjugated edges H of the structural unit, while the electron‐rich regions are distributed across the entire conjugated region (**Figure** **4**a).^[^ ^54^ ^]^ This charge distribution leads to a non‐coincidence of positive and negative charge centers within each structural unit, resulting in the formation of dipoles, which manifests itself as a positively charged edge H and negatively charged conjugate C.^[^ ^41^, ^55^ ^]^ The potential of Cu_3_(HITP)_2_ exhibits uniformly higher negative potentials relative to those noted for Cu_3_(HHTP)_2_ and Cu_3_(THT)_2_, which is again consistent with the extended electronic delocalization in Cu_3_(HITP)_2_. Furthermore, the stronger electronegativity of the ligand atoms results in a substantial potential difference between the metal ions and the ligand atoms, wherein the electrons are more biased toward the ligand atoms. Dipoles are also formed in the direction of the metal ion and the ligand atom. The molecular polarity index (MPI), a quantitative measure derived from the integration and averaging of the electrostatic potential on the molecular surface,^[^ ^56^ ^]^ ranks as follows: 10.33 (Cu_3_(HITP)_2_) > 10.07 (Cu_3_(HHTP)_2_) > 8.26 (Cu_3_(THT)_2_). This ranking is synergistically influenced by the electronegativity of the functionalized ligand motifs and the d‐π conjugation effect (Figure 4b). However, despite the highest electronegativity difference between Cu─X when X = O, Cu_3_(HITP)_2_ has a unique N‐H bond and a more negatively conjugated C greatly enhances the molecular polarity index (Figure S19, Supporting Information).^[^ ^57^, ^58^ ^]^

The more pronounced charge distribution inhomogeneity introduced by the Cu─X bonds units with higher electronegativity difference enables the corresponding MOF layers to possess stronger polarity, which in turn, can offer stronger polarization under external electric field. The electric field distribution simulations based on CST Studio Suite reveal that the field strengths at the rod‐like MOF particles’ centers and vertices are uneven, ascribed to the accumulation of polarization charges at the c‐MOF particle vertex (Figure 4c) (details in the Experimental Section). Specifically, Cu_3_(HITP)_2_ exhibits a stronger polarization field, correlating positively with its MPI value. As the electromagnetic field phase changes, dipoles undergo repeated polarization, causing a reorganization of polarization charges.^[^ ^59^, ^60^ ^]^ This reconfiguration generates a new internal electric field, enabling the conversion of electromagnetic energy into heat through polarization losses, thereby contributing to the attenuation of electromagnetic waves.

Predicated upon the Debye theory, dielectric properties of materials are amenable to be evaluated using Cole‐Cole plots (Equation S1, Supporting Information). The Cole‐Cole diagrams indicate that different structural units of Cu‐X_4_ (O, N, S) exhibit distinct polarization relaxation processes (Figure S21, Supporting Information). Cu_3_(HITP)_2_ elicits multiple semicircles, suggesting its strongest polarization capability, and in agreement with the electric field distribution simulation results. In order to discuss the polarizable nature of MOFs under electric field, the contributions of different spatial positions to the total polarizability are visualized through analyzing the hyperpolarizability density (Figure 4d; Figure S22, Supporting Information).^[^ ^61^, ^62^ ^]^ Cu_3_(HITP)_2_ exhibits an obvious enhancement of the hyperpolarizability density (Table S3, Supporting Information). Cu_3_(HITP)_2_ demonstrates a higher electrode polarization ability, consistent with the results of the Cole‐Cole plot. Additionally, both Cu_3_(HITP)_2_ and Cu_3_(HHTP)_2_ exhibit extended tail lines in the Cole‐Cole plots, indicating significant conduction losses. When EMW interacts with the absorbing material, a higher conductivity will facilitate electron migration and hopping within the material, resulting in localized microcurrents and pronounced conduction losses.^[^ ^63^ ^]^ The slopes of the tail lines indicate that the conduction loss of Cu_3_(HITP)_2_ is greater than that of Cu_3_(HHTP)_2_ due to the superior interlayer and intralayer charge transport capability. This observation is corroborated by computational calculations of the charge transport properties, where Cu_3_(HITP)_2_ was found to reveal superior conductivity near the Fermi level (Figure 4e).^[^ ^64^ ^]^ In contrast, Cu_3_(THT)_2_ does not exhibit a tail line in its Cole‐Cole plot, since the interlayer stacking configuration hinders electron transport between layers, resulting in reduced conductivity and minimal conduction losses.

To evaluate the relative contributions of conduction and polarization losses, average values for each loss mechanism were determined using nonlinear least squares fitting (Figure 4f) (Equations (5), (6), (7)).^[^ ^31^ ^]^ The results reveal that conduction loss is the predominant loss mechanism in the Cu‐X_4_ based c‐MOFs upon EMW exposure. Indeed, Cu_3_(HITP)_2_ exhibits the strongest polarization loss and conduction loss. Because modifying the Cu‐X_4_ coordination bonds is able to facilitate concurrent adjustments in charge transport properties and dipole polarization strength, precise dual‐tuning of the dielectric characteristics is realized. However, Cu_3_(HHTP)_2_ and Cu_3_(THT)_2_ possess relatively low conduction loss due to the locally concentrated charge distribution or ineffective interlayer properties, leading to blocked intra‐ or interlayer charge transport.

**Figure 5:**
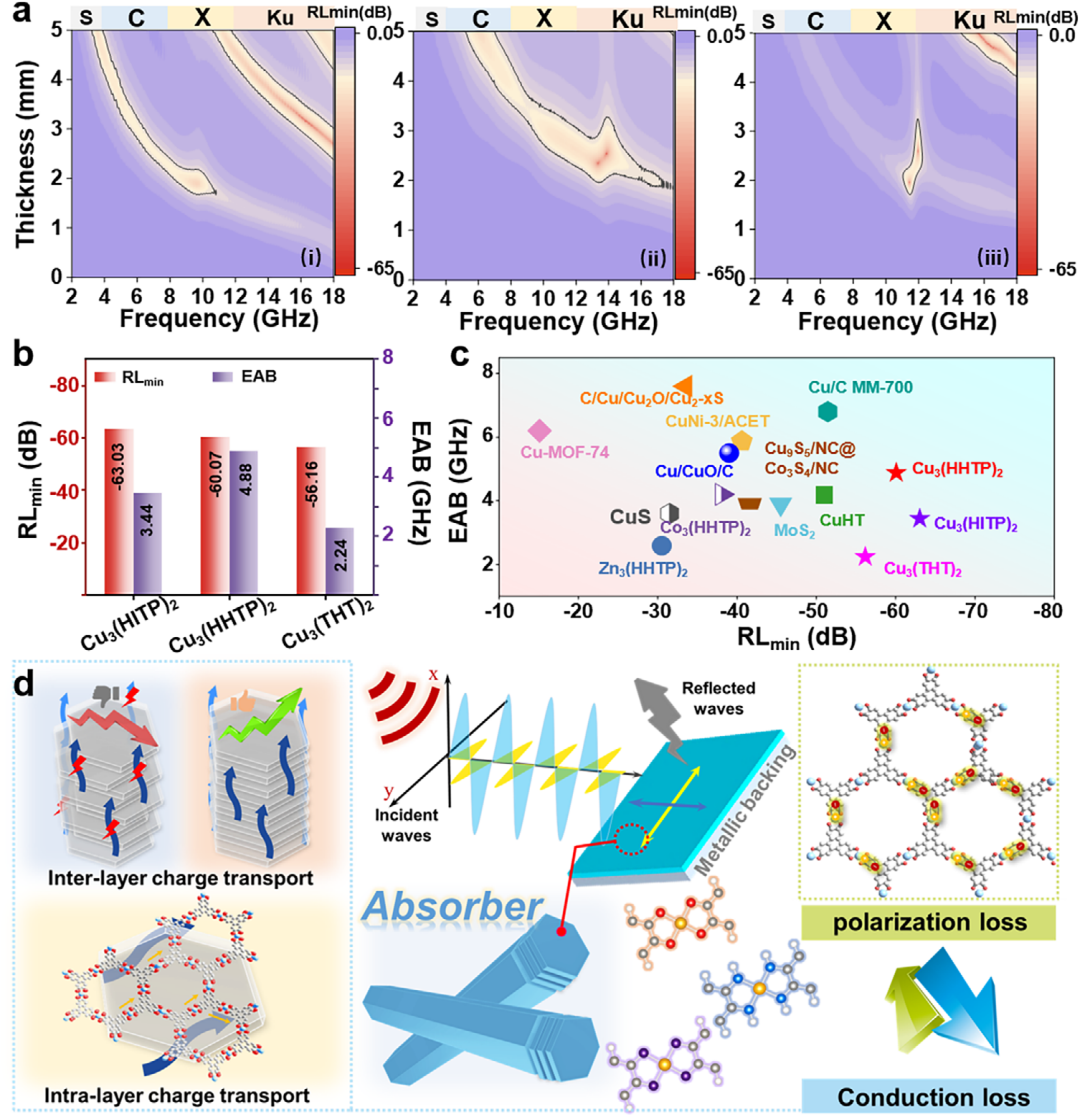
Electromagnetic wave absorption performance. a) The 2D projection of the corresponding reflection loss (RL) values of Cu_3_(HITP)_2_ (i), Cu_3_(HHTP)_2_ (ii), and Cu_3_(THT)_2_ (iii). The portion enclosed by the grey line is the portion with RL_min_ < 10 dB. b) RL_min_ values and maximum effective absorption bandwidth (EAB) values obtained for Cu_3_(HITP)_2_, Cu_3_(HHTP)_2_, and Cu_3_(THT)_2_. c) The comparison of RL_min_ and EAB for c‐MOFs and other Cu‐MOF derivatives‐based EMW absorption materials. d) Schematic illustration of the EMW absorption mechanism realized in the current report.

Vector Network Analyzer measurements (thickness‐frequency‐reflection loss correspondence) show that Cu_3_(HITP)_2_ can achieve effective absorption across the full frequency range (3–18 GHz) by adjusting its thickness, with a minimum reflection loss (RLmin) of ‐63.03 dB at a thickness of 3.2 mm and a maximum effective absorption bandwidth (EAB) of 3.44 GHz (**Figures** **5**a‐i and S27 and S28, Supporting Information) (Equations 1 and 2) Cu_3_(HHTP)_2_ features a RLmin value of ‐60.07 dB and an EAB of 4.88 GHz, but it is less effective in the lower frequency range (Figure 5a‐ii). Cu_3_(THT)_2_ mainly absorbs higher frequencies, with a RLmin value of ‐56.16 dB, and an EAB of only 2.24 GHz (Figure 5a‐iii) (Figure 5b; Figure S26, Supporting Information). All matched thicknesses are within rational boundaries.^[^ ^65^ ^]^ The impedance matching feature plays an essential role in promoting EMW absorption (especially EAB).^[^ ^66^ ^]^ As shown in Figure S25 (Supporting Information), Cu_3_(HHTP)_2_ shows a large impedance matching region, while Cu_3_(HITP)_2_ has a relatively narrow impedance matching region. The interference disruption testified by the 1/4λ theory is further applied to better understand the phenomenon (Figure S25, Supporting Information).^[^ ^67^ ^]^ The same trend was observed for different filling ratios. When comparing Cu‐X_4_ c‐MOFs with other Cu‐MOFs and other conductive MOFs, that Cu‐X_4_ MOFs exhibit a significant advantage in EMW absorption efficiency is evident (Figure 5c; Table S4, Supporting Information). Practical utility of these c‐MOFs were further confirmed through radar scattering cross‐section far‐field simulations (Figures S29 and S30, Supporting Information).

Conduction loss performance is primarily governed by two factors: 1) the strength of d–π conjugation induced by Cu─X (X═N, O, S) coordination, and 2) the stacking arrangement of the molecular layers. The optical bandgap determined from both ligand‐metal and ligand‐ligand levels agrees with the calculated density of states and carrier mobility, reinforcing that Cu_3_(HITP)_2_ linked by Cu─N bonds has the highest intra‐ and interlayer charge transport capacity. Enhanced d–π conjugation facilitates efficient in‐plane charge delocalization, while optimized π‐stacking reduces interlayer transport barriers — together determining the overall electrical conductivity and electromagnetic energy dissipation efficiency. Therefore, Cu_3_(HITP)_2_ has the strongest loss capability among the three studied c‐MOFs, wherein conduction loss transpired as the main mechanism. The enhancement originates from the excellent charge transport channel formed by the d−π conjugation effect of the Cu‐N_4_ unit. Of particular importance is the formation of multiple dipoles by localized negative charge concentration facilitated by the electronegativity differences among Cu−X (Cu−N; Cu−O; Cu−S). The dipoles introduce polarization relaxation under EM field to further enhance the EMW absorption performance. The differential charge density and electrostatic potential elucidate the charge distribution of the c‐MOF monolayer. Cu_3_(HITP)_2_ exhibits a molecular polarization index as high as 10.33, and the polarizability density distribution proves that Cu_3_(HITP)_2_ showcases a higher electrode polarizability (Figure 5d). Thus, for 2D layered conductive MOFs with similar structures and charge transport pathways, the electronegativity differences among atoms in the Cu–X₄ coordination bonds are the primary factors influencing polarization loss. The d–π conjugation effect also plays a key role, as the electrostatic potential gradient between the conjugated carbon atoms of the ligand and terminal hydrogen atoms further enhances polarization. Compared with the metal ion modulation strategy, ligand engineering solves the problem of framework collapse that may be caused by changes in the number of metal matches, and is more conducive to maintaining the topology of the metal nodes for the purpose of fine‐tuning the chemical environment of the coordination. Moreover, abrupt changes in the electronic structure of metals are prone to triggering nonlinear responses and limiting multi‐mechanism synergies, whereas the ligand atoms can realize linear asymptotic modulation through the electronegativity and spatial site resistance.

## 3 Conclusion

The Cu‐X_4_‐composed c‐MOFs (X═N, O, S) coordination environment was critically interrogated, leveraging a prototypical linker modularity‐guided bottom‐up control. Taking the d−π conjugation effects and electronegativity differences of the ligands into cognizance, the metal‐ligand bond environment can act as modulators for tailoring the intra‐planar charge distribution. This is pivotal to the intra‐ /inter‐layer charge transport in the c‐MOFs, which further regulate their EM‐simulated polarization and conduction loss. The excellent d−π conjugation effect of Cu —NH enables highly efficient intra‐ and interlayer charge transport channels, optimizing the intensity of in‐plane dipole polarization (conjugated C with edge H, Cu─N and N —H). Cu_3_(HITP)_2_ achieves a prominent RL_min_ up to ‐63.03 dB, and realizes a wide absorption bandwidth of near‐complete absorption over the entire frequency band (3–18 GHz) through thickness tuning. Moving beyond this first example, the developed ligand regulation strategy has the potential to serve as a new reference blueprint for the design of molecular EMW absorber materials in the future.

## 4 Experimental Section

### Materials

Cupric acetate monohydrate [Cu(OAc)_2_·2H_2_O], Copper sulfate monohydrate [CuSO_4_·5H_2_O], Copper(II) trifluoroacetylacetonate, 2,3,6,7,10,11‐hexahydroxytriphenylene (HHTP), Hexaaminotriphenylene hexahydrochloride (HITP·6HCl), 2,3,6,7,10,11‐Hexathiotriphenylene (THT), Sodium acetate (NaOAc), Deionized water, Acetone, N,N‐dimethylacetamide (DMAC), Methanol, N,N‐dimethylformamide (DMF), Ethanol, Ammonium hydroxide. Organic ligands are purchased from Bidepharm, Copper source purchased from Aladdin Inc, Solvents produced by Macklin, Standard for GC,>99.9%.

### Synthesis of Cu_3_(HHTP)_2_

The HHTP (260 mg, 0.8 mmol) is dispersed by sonication in 125 mL of DI‐Water (10 min). Cu(OAc)_2_·H_2_O (320 mg, 1.6 mmol) is dissolved in 100 mL of DI‐Water. The metal salt solution is then added dropwise to the aqueous dispersion of HHTP and sonicated for 15 min. Subsequently, the mixture is stirred continuously for 15 h at 85 °C. The reaction proceeds in an air environment in order to fully oxidize the ligand and avoid reduction of metal ions. The blue‐black precipitate was separated by filtering after the reaction solution was cooled to room temperature. The precipitate was washed three times alternately with H2O and methanol. Finally, the product is dried in a vacuum oven at 60 °C for 12 h.

### Synthesis of Cu_3_(HITP)_2_

The HITP·6HCl (240 mg, 0.446 mmol) is dispersed by sonication in 72 mL of DI‐water (10 min). CuSO_4_·5H_2_O (162 mg, 0.672 mmol.) is dissolved in 72 mL of DMA and sonicated for 10 min. The metal salt solution is then added to the aqueous dispersion of HITP and sonicated for 10 min. Subsequently, 32 mL of NaOAc aqueous solution (2 mol/L) is added and the mixture is heated with stirring at 65 °C for 32 h. The brown‐black precipitate was separated by filtering after the reaction solution was cooled to room temperature. The precipitate was washed three times alternately with H_2_O and methanol. Finally, the product is dried in a vacuum oven at 60 °C for 12 h.

### Synthesis of Cu_3_(THT)_2_

The THT (200 mg, 0.48 mmol) is dispersed by sonication in 15 mL of DI‐water (degassed with N_2_ and adjusted to pH = 10 by 300 µL of 28% ammonium hydroxide). Copper (II) trifluoroacetylacetonate (355 mg, 0.96 mmol) is dissolved in 15 mL of DI‐water (degassed with N_2_). The metal salt solution is then added to the aqueous dispersion of THT and sonicated for 10 min. The mixture was stirred at 65 °C for 24 h under N_2_. The black precipitate was separated by filtering after the reaction solution was cooled to room temperature. The precipitate was washed three times alternately with H2O and acetone. Finally, the product is dried in a vacuum oven at 60 °C for 12 h.

### Materials Characterization

The morphology and microstructure were characterized by a scanning electron microscope (SEM; SU‐70) and High‐resolution transmission electron microscopy (HRTEM; JEM 2100F). Energy dispersive X‐ray spectroscopy (EDS) was used for elemental analyses, equipped with a Bruker Edax light‐element Si (Li) detector (Billerica, MA). The X‐ray diffractometer with Cu‐Kα radiation (XRD; Bruker D8ADVANCE) was used to examine crystal structures over a 2θ range of 2° to 50°. The FTIR (Thermo Scientific Nicolet Is20) spectra were recorded to analyze the functional group. X‐ray photoelectron spectroscopy (XPS) was carried out (Sigma Probe, PHI QUANTERA II) to analyze the chemical state of samples. N_2_ adsorption and desorption of MOFs were performed by Brunner‐Emmet‐Teller (MicroActive for ASAP 2460 2.01). Pore size distributions and pore volumes were derived from the adsorption isotherms. The thermogravimetric curves from 30 to 900 °C at a heating rate of 10 °C per minute under nitrogen atmosphere were obtained by Thermal Gravimetric Analyzer (DSC‐TGA Standard, SDT Q600 V20.9 Build 20). Raman spectra were collected on a Raman spectrophotometer (Aramis) with a 532 nm solid laser as an excitation source (500 ∼ 2500 cm^−1^). Electron paramagnetic resonance (EPR) measurements were performed using Bruker EMXplus, a spectrometer equipped with a standard mould cavity and a liquid nitrogen cryostat system. Vibrating Sample Magnetometer (VSM, LakeShore7404) Measured Saturation Magnetization Intensity. Four‐point probe (RTS‐8) was used to research the electrical conductivity (EC) of samples. The electrochemical performance was detected by a CHI 660E electrochemical workstation. A three‐electrode cell configuration was employed, and all samples should be coated on a glassy carbon electrode as working electrodes. Additionally, a saturated silver chloride electrode, a Pt wire, and 1 m NaCl aqueous solution were employed as reference electrode, counter electrode, and electrolyte, respectively. Cu K‐edge XAFS analyses were performed at the BL14W Beam‐line at the Shanghai Synchrotron Radiation Facility (SSRF) (Shanghai, China).

### Electromagnetic Measurement

The vector network analyzer (Keysight N5222B) is utilized to measure electromagnetic parameters containing complex permittivity and permeability. Prior to this, all samples (60 wt.%) mixed homogeneously with paraffin were pressed into rings in paraffin (Φin = 3.04 mm, Φout = 7.00 mm). According to the transmission line theory in the metallic backing condition, The EMA performance at a specific frequency (f) and absorber thickness (d) can be calculated according to the following Equations (1) and (2):^[^ ^68^ ^]^

(1) $$RL\left(dB\right)=20log_{10}\frac{\left|Z_{in}-Z_{0}\right|}{\left|Z_{in}+Z_{0}\right|}$$

(2) $$Z_{in}=Z_{0}\sqrt{\frac{\mu_{r}}{\varepsilon_{r}}}\tanh\left(j\frac{2\pi fd}{c}\sqrt{\mu_{r}\varepsilon_{r}}\right)$$

where Z_in_, Z_0_, f, d and c represent the impedance of absorbers, the impedance of free space, incident EMW frequency, thickness of absorbers and velocity of light.

### RCS Simulations

The CST Studio Suite 2020 software was utilized to perform the RCS simulation using the metal backplane and Predator II as models, respectively.

For the metal backplane, model construction and excitation configuration: the width of the perfect electric conductor (PEC) plate was 50.0 × 50.0 mm^2^ with a thickness of 5.0 mm, the plate surface coated with a certain thickness of MOF absorbing layer. A single‐frequency plane wave is selected as the excitation source. For the setting of polarization waves, the incident azimuth angles were restricted within the “‐90° ≤ φ ≤ 90°,θ = 30°, 60°, 90° ” condition. The polarization mode was linear. When the incident wave varies with azimuth and the direction of the electric field is always parallel to the plate, the incident wave is called a horizontally polarized wave, and vice versa for a vertically polarized wave. The RCS values can be described as follows^[^ ^69^ ^]^:

(3) $$\sigma \left(dBm^{2}\right)=10\log{\left(\frac{4\pi S}{\lambda^{2}}\left|\frac{E_{s}}{E_{i}}\right|\right)}^{2}$$

Similarly, for Predator II, model construction and excitation configuration: The Predator II warbird constructed from PEC material is coated with a certain thickness of MOF absorbing layer. A single‐frequency plane wave is selected as the excitation source (the frequency of the strongest EMW absorption). the incident azimuth angles were restricted within the “0° ≤ φ ≤ 360°, θ = 90° ” condition. The polarization mode was linear.

### Electric Field Distribution Simulation

The electric field distribution of c‐MOF was simulated by CST Studio Suite to clarify the polarization behavior of hexagonal prismatic structure. During the simulation, the electromagnetic properties of derivatives, including the real parts of complex permittivity were set according to the experimental data. Finally, the driven solution frequency was set as 10 GHz while the strength and direction of applied electric field were set to 1V·m^−1^ and vertical downward, respectively.

### DFT Simulations

All calculations were conducted using first‐principles methods based on density functional theory (DFT), as implemented in the Vienna Ab Initio Simulation Package (VASP) code.^[^ ^70^, ^71^ ^]^ The Perdew‐Burke‐Ernzerhof (PBE) exchange‐correlation functional was employed for the DFT calculations.^[^ ^72^ ^]^ A kinetic energy cutoff of 400 eV was chosen based on convergence tests. The self‐consistent total energy convergence criterion was set to 10⁻⁶ eV. For k‐space sampling, a 5 × 5 × 1 k‐point mesh was generated using the Monkhorst‐Pack scheme to balance computational cost and precision.

Carrier mobility calculation: The carrier mobility is calculated based on the deformation potential theory, and the formula is as follows:^[^ ^73^ ^]^

(4)

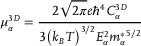




Here, e represents the elementary charge; ℏ is the reduced Planck constant; $C_{\alpha }^{3D}$ denotes the elastic constant; T represents the temperature; E_α_ is the deformation potential energy; and m^∗^ represents the effective mass of the carrier. In addition, the model incorporates metal substitution at specific copper sites to calculate the nature of charge transport at different metal centers.

### Non‐Linear Least Squares Fitting

Conductive loss (ε_c_“) and polarization loss (ε_p_”) values were fitted to each compliance using Non‐linear least squares via running Python. Non‐linear least squares fitting is a classic and precise way to fit a curve. According to Debye theory, the model function in this work as follows:[^62^]

(5) $$\varepsilon =\varepsilon_{\infty }+\frac{\left(\varepsilon_{s}-\varepsilon_{\infty }\right)}{\left(1+\omega^{2}\tau^{2}\right)}-i\left[\frac{\left(\varepsilon_{s}-\varepsilon_{\infty }\right)\omega \tau }{\left(1+\omega^{2}\tau^{2}\right)}+\frac{\sigma }{\left(\omega \varepsilon_{0}\right)}\right]$$

Among them,

(6) $$\varepsilon^{\prime }=\varepsilon_{\infty }+\frac{\left(\varepsilon_{s}-\varepsilon_{\infty }\right)}{\left(1+\omega^{2}\tau^{2}\right)}$$

(7)

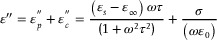

where ε_∞_ is optical dielectric constant, ε_*s*_is static dielectric constant, ε_0_ is free space dielectric constant, τ is the relaxation time, and σ is the conductivity. The parameters needed to fitted are ε_∞_, ε_*s*_, ε_0_, τ and σ, which are signed as a group β. The sum of squares is $S=\sum_{i=1}^{m}r_{i}^{2}$, where *r_i_* = ε_*fit*_  − ε. Then the goal is minimizing the S by adjusting β. The mean number is the result.

## Conflict of Interest

The authors declare no conflict of interest.

## Supporting information



Supporting Information

## Data Availability

The data that support the findings of this study are available from the corresponding author upon reasonable request.
