# Supplementary material for: Cu─X Bonds Regulated Conduction and Polarization Loss in Conductive Metal‐Organic Framework Under Electromagnetic Field
Source: Adv Sci (Weinh). 2025 Jun 10;12(33):e08379. doi: 10.1002/advs.202508379 (PMC12412561; doi:10.1002/advs.202508379)
Supplement: Supplementary file 1 — Supporting Information [file ADVS-12-e08379-s001.docx]

Supporting Information

Cu-X Bonds Regulated Conduction and Polarization Loss in Conductive Metal-Organic Framework under Electromagnetic Field

Siyao Cheng,^†^ Qinglin Zhou,^†^ Daohu Sheng, Wei Dong, Jinhu Dou, Yuanbiao Huang, Rong Cao, Aming Xie,^*^ Roland A. Fischer, Soumya Mukherjee, and Weijin Li^*^

Dr. S. Y. Cheng, Prof. W. J. Li

MIIT Key Laboratory of Advanced Display Materials and Devices & Materials Physical and Chemical Research and Practice Center, College of Materials Science and Engineering, Nanjing University of Science and Technology, Nanjing, 210094 China
E-mail: wjli@njust.edu.cn

Dr. S. Y. Cheng, Prof. Y. B. Huang, Prof. R. Cao
State Key Laboratory of Structural Chemistry, Fujian Institute of Research on the Structure, Chinese Academy of Sciences, Fuzhou 350002, P. R. China

Q. L. Zhou, D. H. Sheng, Prof. W. Dong

School of Chemistry and Chemical Engineering, Nanjing University of Science and Technology, Nanjing 210094, P. R. China

Prof. A. M. Xie

School of Safety Science and Engineering, Nanjing University of Science and Technology, Nanjing 210094, P. R. China

E-mail: xieaming@njust.edu.cn

Prof. S. Mukherjee

Bernal Institute and Department of Chemical Sciences, University of Limerick, Limerick V94 T9PX, Ireland

Prof. J. H. Dou

School of Materials Science and Engineering, Peking University Zonghe Science Building, 700871, P. R. China

Prof. R. A. Fischer,

Chair of Inorganic and Metal-organic Chemistry, Department of Chemistry & School of Natural Sciences, Technical University of Munich, Lichtenbergstrasss 4, 85748, Garching, Germany

^†^Dr. S. Y. Cheng and Ms. Q. L. Zhou contributed equally to this work.

**Content**

[1. Supplementary Figures 4](#_Toc197249458)

[Section 1: S1-S4 SEM/TEM images and EDS spectra of c-MOFs. **4**](#_Toc197249459)

[Section 2: S5 PXRD characterization of bulk of c-MOFs. **8**](#_Toc197249460)

[Section 3: S6 Brunner−Emmet−Teller (BET) measurements of c-MOFs. **9**](#_Toc197249461)

[Section 4: S7 FT-IR curves of c-MOFs. **10**](#_Toc197249462)

[Section 5: S8-S9 XPS characterization of c-MOFs. **11**](#_Toc197249463)

[Section 6: S10-S11 Cu-X bond coordination of c-MOFs. **13**](#_Toc197249464)

[Section 7: S12 Hirshfeld charge transfer of c-MOFs. **16**](#_Toc197249465)

[Section 8: S13 Thermal stability of c-MOFs. **17**](#_Toc197249466)

[Section 9: S14-S15 Electrically conductive properties of bulk of c-MOFs. **18**](#_Toc197249467)

[Section 10: S16 Band structure of monolayer c-MOFs. **20**](#_Toc197249468)

[Section 11: S17 Diffuse reflectance UV-vis-NIR spectra of c-MOFs. **21**](#_Toc197249469)

[Section 12: S18 EPR spectra of c-MOFs. **22**](#_Toc197249470)

[Section 13: S20-S29 Electromagnetic wave absorption properties **24**](#_Toc197249471)

[4. Supplementary Tables 35](#_Toc197249472)

[3. Reference 39](#_Toc197249473)

# 1. Supplementary Figures

## Section 1: S1-S4 SEM/TEM images and EDS spectra of c-MOFs.


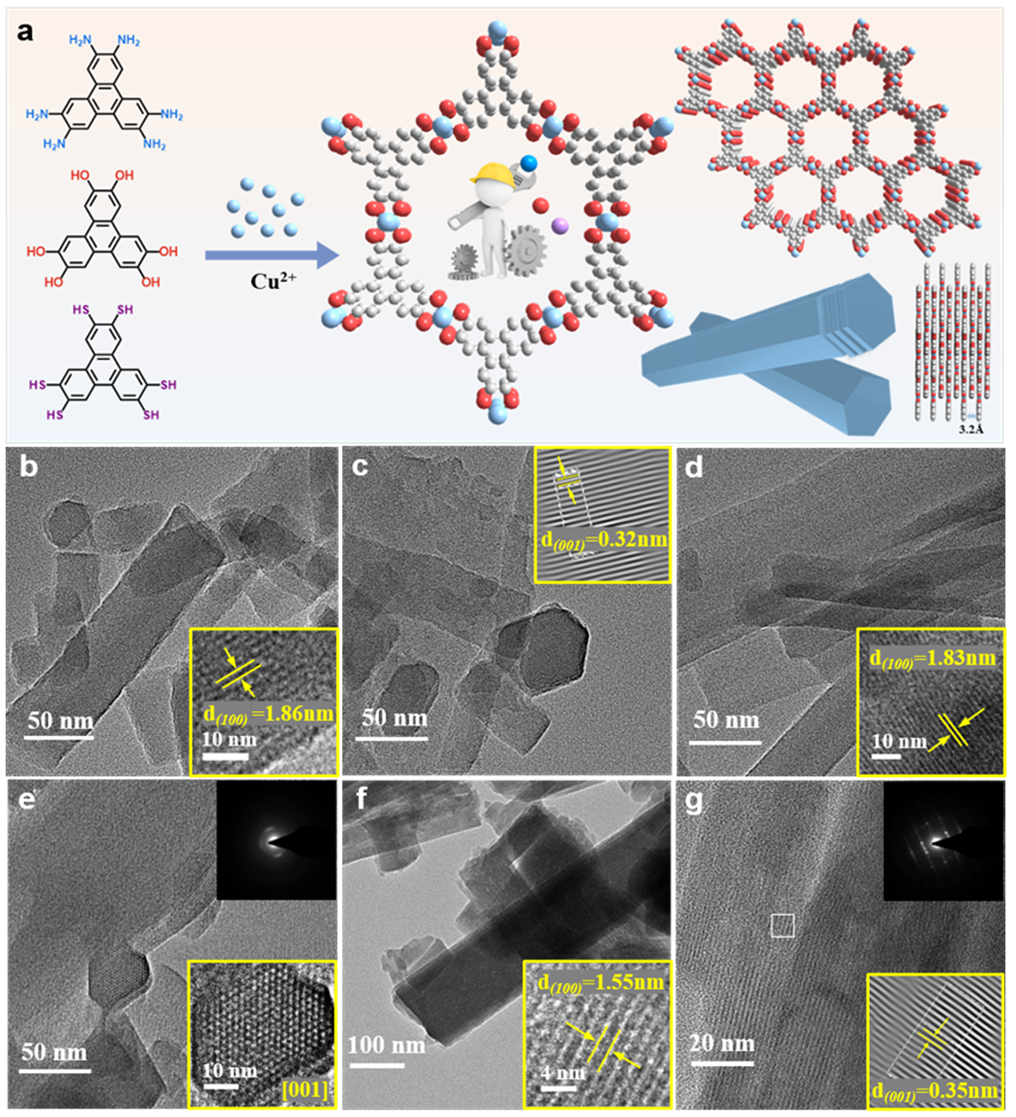


**Figure S1.** Synthesis and characterization. (a) Synthetic illustration and crystal structures of Cu_3_(HITP)_2_, Cu_3_(HHTP)_2_, and Cu_3_(THT)_2_. (b-g) HR-TEM images and electron diffractions (recorded for selected areas) of Cu_3_(HITP)_2_ (b, c), Cu_3_(HHTP)_2_ (d, e), Cu_3_(THT)_2_ (f, g).


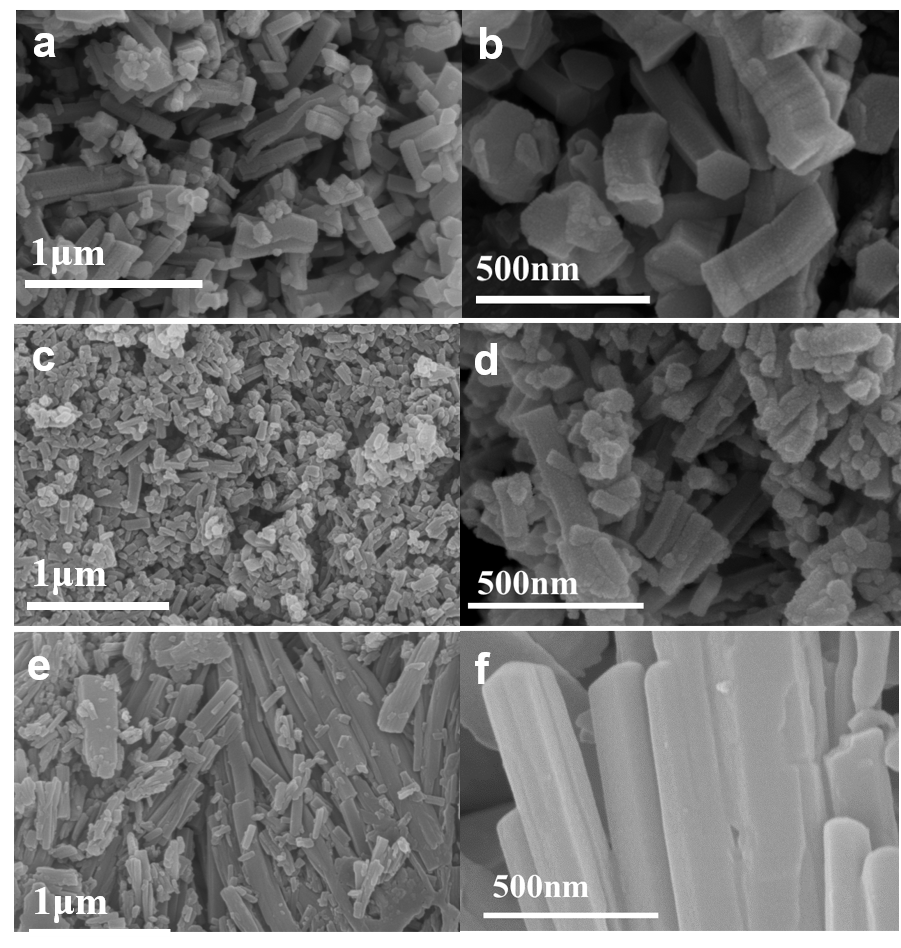


**Figure S2.** The SEM micrographs for (a, b) Cu_3_(HHTP)_2_, (c, d) Cu_3_(HITP)_2_ and (e, f) Cu_3_(THT)_2_.

The crystal morphologies were further confirmed by high-resolution transmission electron microscopy (HR-TEM) images **(Figures S1 and S3)**. As shown in **Figures S1b-g**, all three c-MOFs demonstrate hexagonal channels along the [001] direction. The electron beam incident from the [001] direction results in exposing the axial surface of the hexagonal prism-shaped crystals. A honeycomb network constructed from the hexadentate ligand backbones can also be identified from the HR-TEM images. This signifies the formation of a prototypal conductive network, enabled by the c-MOFs shared characteristic architecture herein. Cu_3_(HITP)_2_ and Cu_3_(HHTP)_2_ demonstrate hexagonal channels with a window size of approximately 1.8 nm, manifested in the (100) crystallographic plane (2θ = 4.6°) **(Figure S1b and 1d)**. Conversely, Cu_3_(THT)_2_ exhibits a lattice spacing of 1.6 nm along the (100) plane (2θ = 5.3°) **(Figure S1f)**. The lattice distances of 3.2 Å and 3.5 Å noted in Cu_3_(HITP)_2_ and Cu_3_(THT)_2_ can be indexed to the interlayer distances of their (201) and (001) crystal planes, respectively (**Figures S1c and 1g**). Scanning electron microscopy (SEM) micrographs reveal nano-hexagonal prism as the uniform polycrystalline morphology, indicating a high degree of extended order in each c-MOF (**Figure S2**).


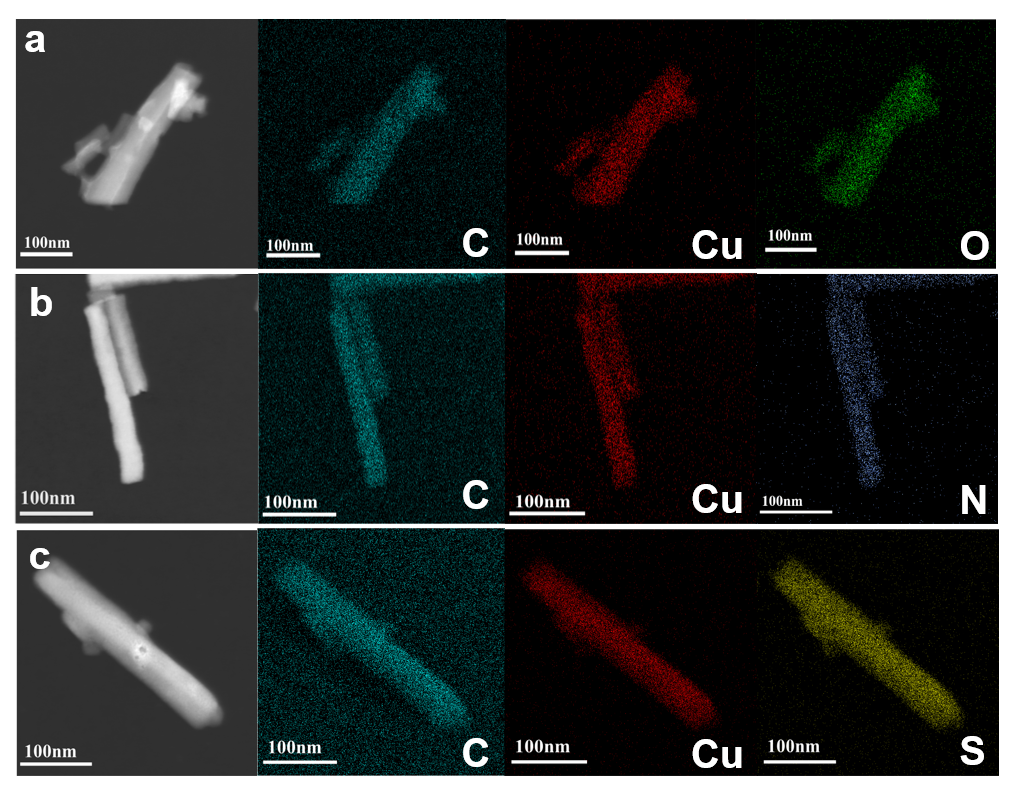


**Figure S3.** The HAADF-STEM micrographs and the corresponding EDS mappings of C, O, N, S and Cu in (a) Cu_3_(HHTP)_2_, (b) Cu_3_(HITP)_2_ and (c) Cu_3_(THT)_2_.


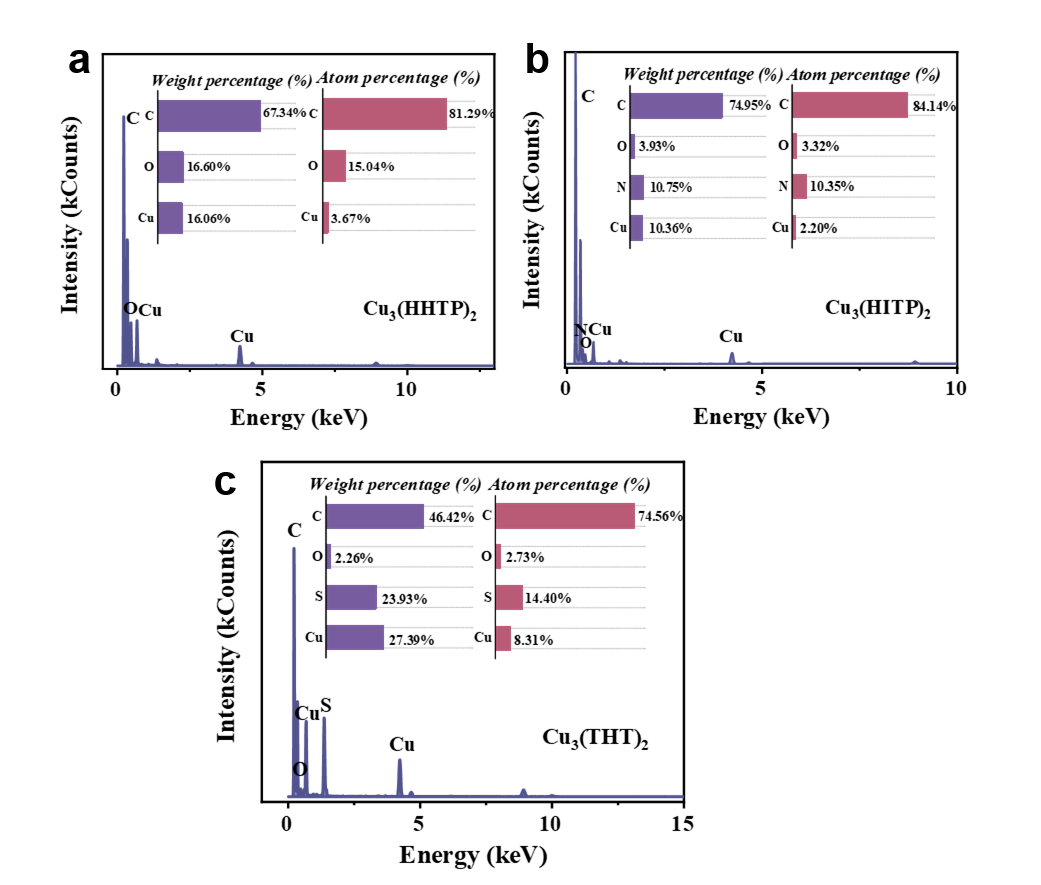


**Figure S4.** EDX spectra and the corresponding elemental statistics for (a) Cu_3_(HHTP)_2_, (b) Cu_3_(HITP)_2_ and (c) Cu_3_(THT)_2_.

## Section 2: S5 PXRD characterization of bulk of c-MOFs.


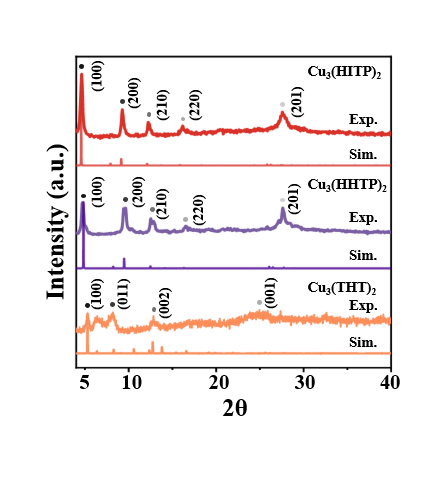


**Figure S5.** PXRD patterns of Cu_3_(HITP)_2_, Cu_3_(HHTP)_2_, and Cu_3_(THT)_2_ (Exp. = Experimental; Sim. = Simulated).

For Cu_3_(HITP)_2_ and Cu_3_(HHTP)_2_, three prominent diffraction peaks were observed at 4.7°, 9.6°, and 12.5° corresponding to the (100), (200), and (210) planes, respectively.^[^[[1]](#endnote-2)^,^[[2]](#endnote-3)^]^ Specifically, the diffraction peak at 27.6° corresponds to the (001) crystal plane, indicating that both Cu_3_(HITP)_2_ and Cu_3_(HHTP)_2_ exhibit a layered structure, characterized by slipped parallel packing of the layers, with an interlayer spacing of *ca*. 3.2 Å.^[^^^[[3]](#endnote-4)^]^ In contrast, Cu_3_(THT)_2_ exhibits prominent peaks at 5.3°, 6.33°, and 12.75°, with an interlayer distance of 3.5 Å, resembling the characteristic signature peaks for Cu_3_(THT)_2_, and thus suggesting staggered packing.^[^^^[[4]](#endnote-5)^,^[[5]](#endnote-6)^]^

## Section 3: S6 Brunner−Emmet−Teller (BET) measurements of c-MOFs.


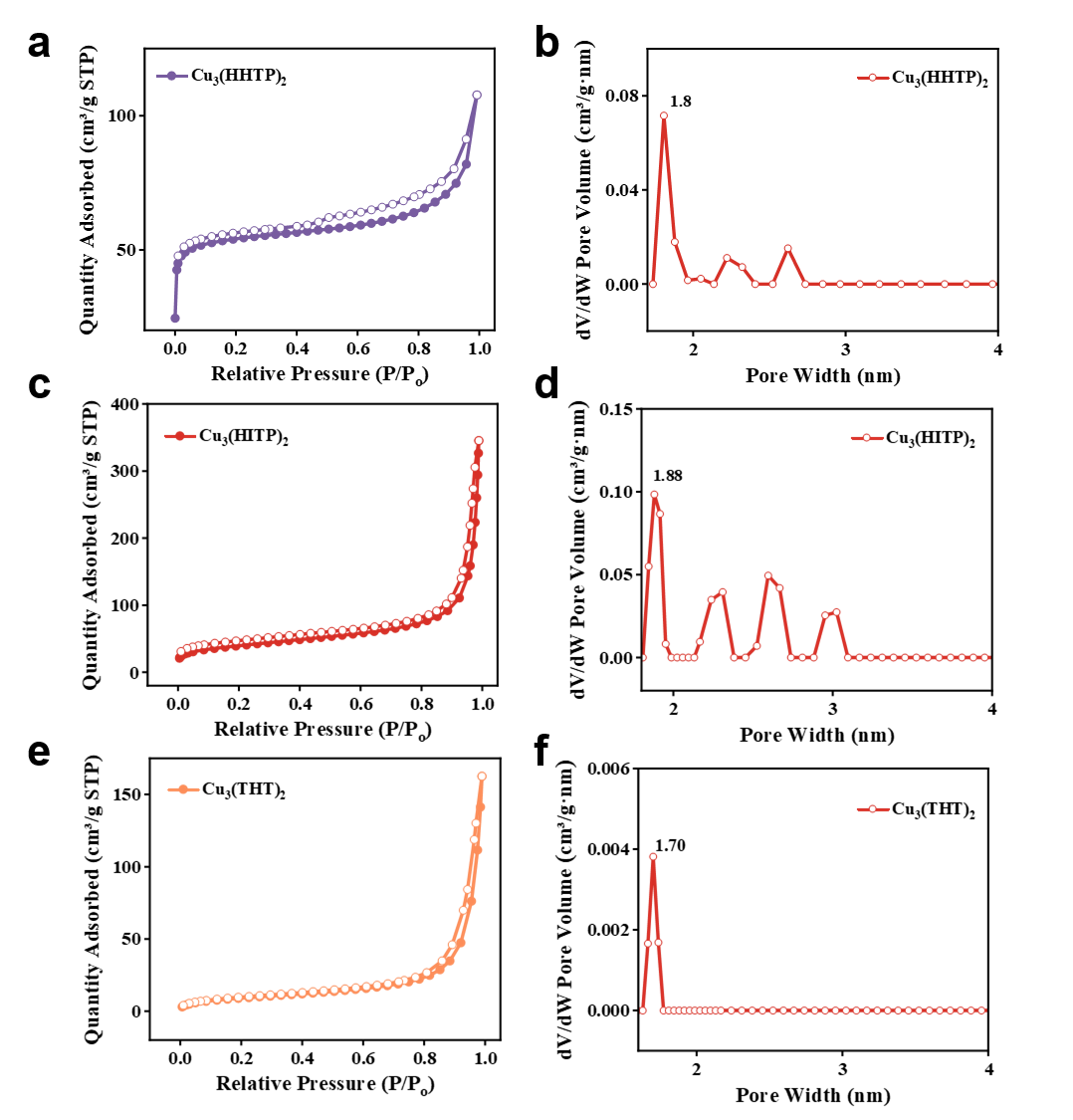


**Figure S6.** The N_2_ adsorption-desorption isotherms and pore size distributions of (a, b) Cu_3_(HHTP)_2_, (c, d) Cu_3_(HITP)_2_ and (e, f) Cu_3_(THT)_2_. Closed and open symbols denote adsorption and desorption, respectively.

Porosities for the MOFs were further determined by nitrogen adsorption analysis at 77 K temperature. According to the results they all exhibited type I isotherms, which is typical of microporous materials. Notably, the specific surface areas of Cu_3_(HHTP)_2_, Cu_3_(HITP)_2_ and Cu_3_(THT)_2_ were 206.5415 m²/g, 232.7815 m²/g and 31.7610 m²/g, respectively. Cu_3_(THT)_2_ possesses smaller pore size and BET specific surface area due to ligand dislocation stacking.

## Section 4: S7 FT-IR curves of c-MOFs.


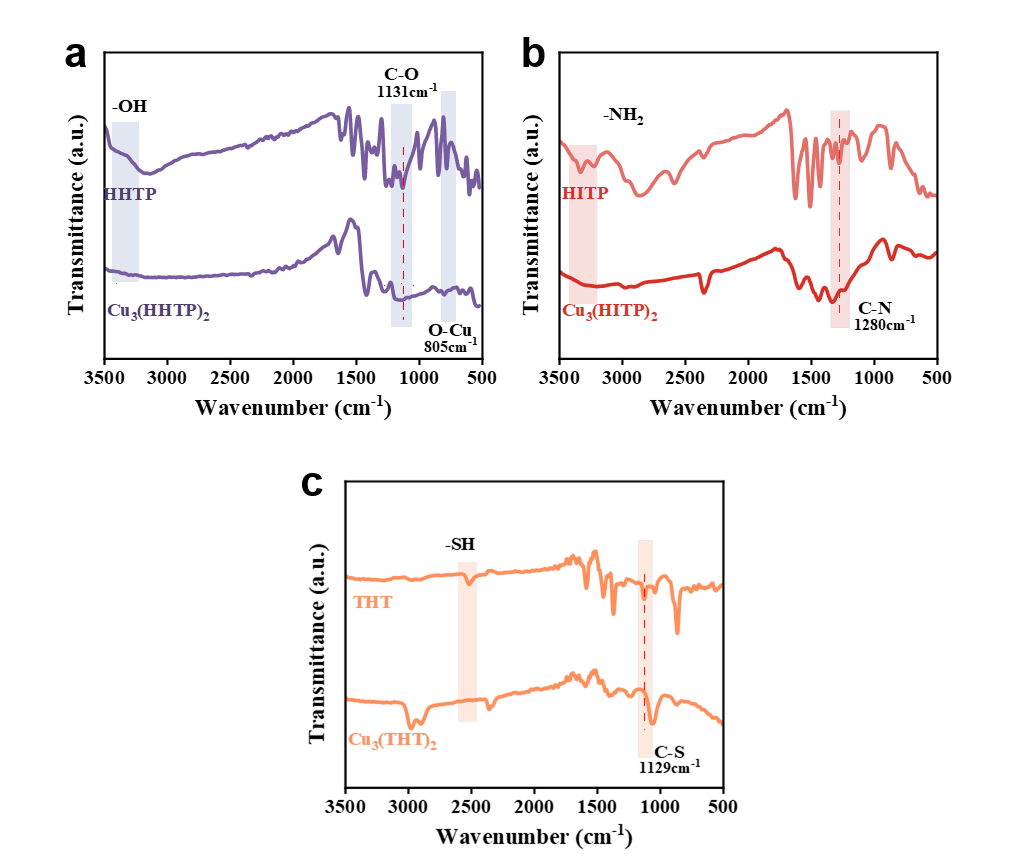


**Figure S7.** The FT-IR spectra of (a) Cu_3_(HHTP)_2_, (b) Cu_3_(HITP)_2_ and (c) Cu_3_(THT)_2_.

The characteristic stretching signals of -NH_2_ (3300-3500 cm^-1^), -OH (3200-3600 cm^-1^), and -SH (2550-2700 cm^-1^) are found to be absent in these ligands-derived c-MOFs’ spectra, reinforcing the complete conversion to Cu-X coordination bonds from the exposed oxygen/nitrogen/sulfur atoms (in the ligands) (**Figure S7**).^[^^^[[6]](#endnote-7)^,^[[7]](#endnote-8)^]^ The relative weakness of C-NH_2_, C-OH, and C-SH FT-IR peak intensities for Cu-X in the c-MOFs *vs*. those in the pristine ligands suggests Cu^(II)^ coordination to the π-conjugated triphenylene-based ligands that enables extended electron delocalization across the c-MOF structures **(Figure S6)**. Such electron delocalization is known to benefit intralayer charge transport. ^[^[[8]](#endnote-9)^]^

## Section 5: S8-S9 XPS characterization of c-MOFs.


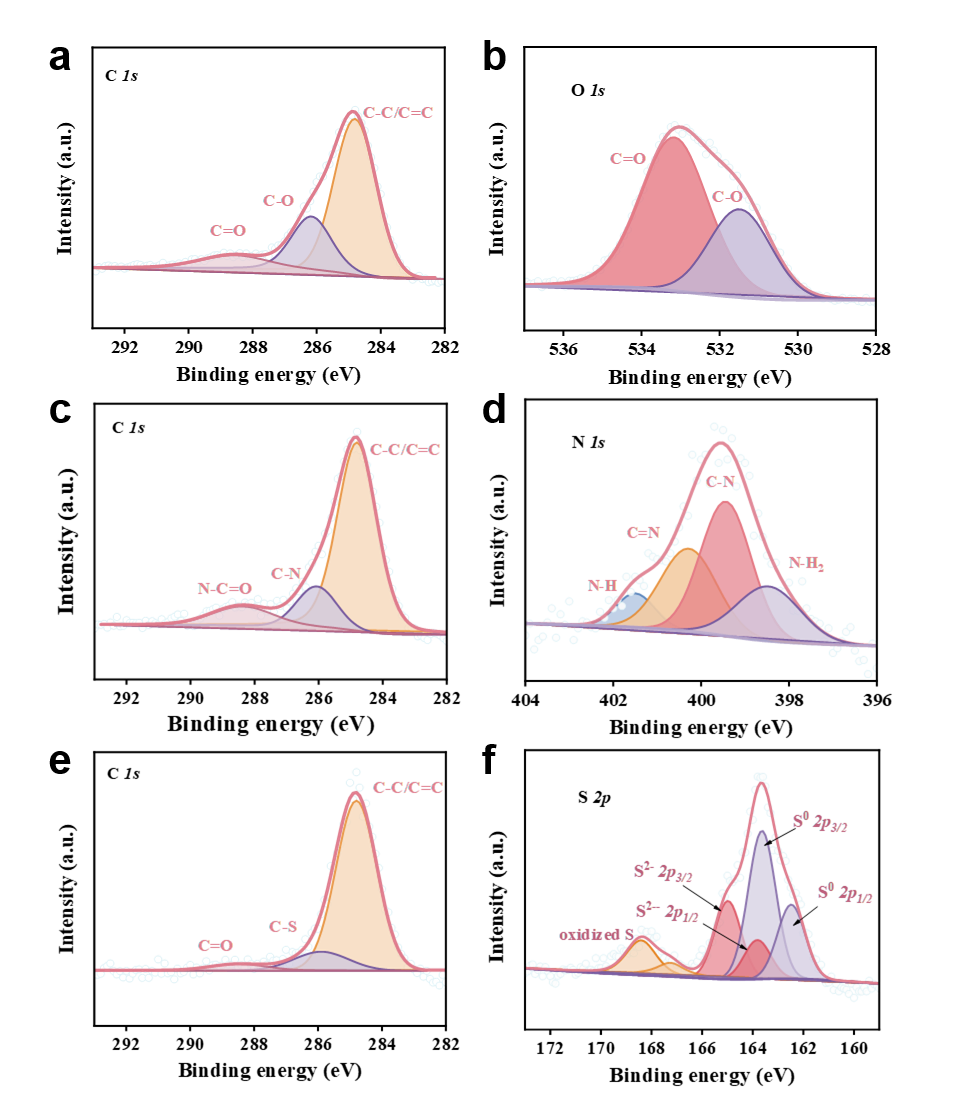


**Figure S8.** XPS spectra: The C *1s* of (a) Cu_3_(HHTP)_2_, (c) Cu_3_(HITP)_2_ and (e) Cu_3_(THT)_2_. (b) The O *1s* of Cu_3_(HHTP)_2_. (d) The N *1s* of Cu_3_(HITP)_2_. (f) The S *2p* of Cu_3_(THT)_2_.

As is shown in **Figure S8**, The C *1s* peak of Cu_3_(HHTP)_2_ can be deconvoluted into three parts at 284.8, 286.2, and 288.6 eV, corresponding to the chemical states of C-C/C=C, C-O, and C=O. The C-C/C=C peaks originate from the π-conjugated benzene ring, whilst the presence of C=O reasonably demonstrates that the ligand hydroxyls undergo deprotonation and oxidation in the coordination process. [^[[9]](#endnote-10)^] Similarly, C-O (531.4 eV) and C=O (533.1 eV) were observed in O1s region. The C-N in Cu_3_(HITP)_2_ and the C-S in Cu_3_(THT)_2_ correspond to the peak at 286.1 eV and 285.9eV in the deconvolution of the C *1s* region. The prevalence of C=O in C *1s* region indicates that the MOFs are all oxidized to different degrees. Therefore, the double peaks located at 168.4 eV and 167.3 eV in the S*2p* spectrum of Cu_3_(THT)_2_ are attributed to the oxidation state S. The deconvolution peaks in the S*2p* region give rise to two sets of double peaks in addition to the oxidation state S described above, those at 165.0 eV and 163.8 eV belonging to in-frame S coordinated to Cu (Cu-S), while the double peaks at 163.6 eV and 162.5 eV with high intensities belong to the partially uncoordinated thiol (S-H).^[^^[[10]](#endnote-11),^^[[11]](#endnote-12)]^ The N *1s* region indicates the presence of four different chemical environments (398.5 eV, 399.4 eV, 400.3 eV, 401.5 eV) for the nitrogen of Cu_3_(HITP)_2_, and the peak at 400.3 eV shows coordination N (N-Cu) in the MOF, the presence of N-H_2_ at 398.5 eV indicates the presence of amino groups in the MOF that are not involved in coordination.


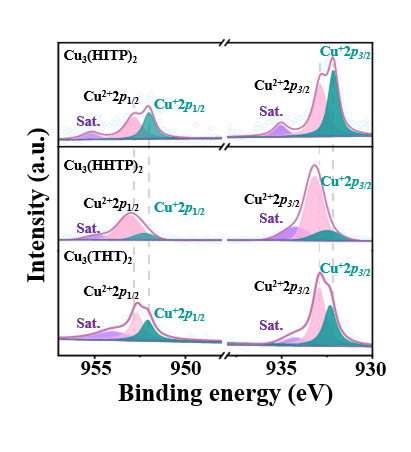


**Figure S9.** XPS Cu 2*p* spectra of Cu_3_(HITP)_2_, Cu_3_(HHTP)_2_, and Cu_3_(THT)_2_.

The Cu *2p* spectra of Cu_3_(HITP)_2_, Cu_3_(HHTP)_2_, and Cu_3_(THT)_2_ exhibit mixed valence states for Cu^(II)^ and Cu^(I)^, indicating that during synthesis, deprotonation and oxidation of the ligands led to the partial reduction of Cu^(II)^ to Cu^(I)^. Predicated upon the integrated peak areas, the Cu^(II)^/Cu^(I)^ ratios were determined to follow the increasing order: 1.06 (Cu_3_(HITP)_2_) < 1.37 (Cu_3_(THT)_2_) < 4.18 (Cu_3_(HHTP)_2_) (**Table S1**). Compared to the Cu *2p_1/2_* ^(II)^ peak in Cu_3_(HHTP)_2_ at 953.1 eV, the Cu *2p_1/2_* ^(II)^ peaks in Cu_3_(HITP)_2_ and Cu_3_(THT)_2_ were found to be shifted by 0.2 eV and 0.4 eV towards lower binding energies, respectively. This peak shift indicates that the degree of electron transfer from Cu^(II)^ to O atoms is stronger relative to the S and N atoms, likely related to the three heteroatoms’ different electronegativities.^[^14^,^[[12]](#endnote-13)^]^ Stronger electronegativity of X (such as, in O) leads to pronounced concentration of electrons on it, resulting in a higher Cu-X charge separation, optimizing the dipoles thereof. On the flipside, localized concentration of electrons culminates in an uneven charge distribution in the c-MOF plane, thus contributing negatively to charge transport.

## Section 6: S10-S11 Cu-X bond coordination of c-MOFs.


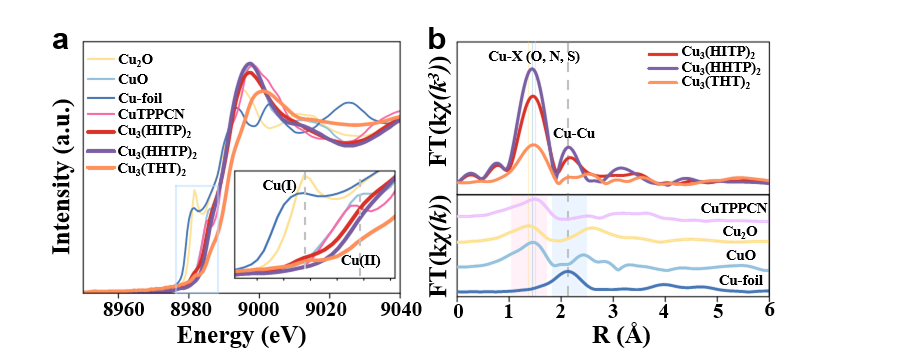


**Figure S10.** Normalized Cu K-edge XANES spectra of Cu_3_(HITP)_2_, Cu_3_(HHTP)_2_, and Cu_3_(THT)_2_ samples with respect to CuO, Cu_2_O, Cu-foil and CuTPPCN standard. (d) Comparison of Fourier transforms of the EXAFS oscillation at Cu K-edge for Cu_3_(HITP)_2_, Cu_3_(HHTP)_2_, and Cu_3_(THT)_2_samples concerning CuO, Cu_2_O, Cu-foil and CuTPPCN (Cu-N4 standard structure) standards. CuTPPCN =Meso-Tetra (4-cyanobenzyl) porphine copper(II). ^[^^[[13]](#endnote-14)]^

Electronic structures and coordination environments of the prepared c-MOFs were further investigated using X-ray absorption fine structure spectroscopy (EXAFS). The normalized X-ray absorption near-edge fine structure (XANES) spectra of Cu_3_(HITP)_2_ at the Cu K-edge reveal a shoulder peak at 8982 eV, which belongs to the characteristic peak of Cu^(I)^. The shoulder at 8986 eV is assigned to the characteristic Cu^(II)^ peak **(Figure S10a)**. ^[^[[14]](#endnote-15)^,^[[15]](#endnote-16)^]^ The Cu^(I)^ signal was found to be exceptionally pronounced in Cu_3_(HITP)_2_. The Cu K-edge extended EXAFS spectra were transformed by Fourier transforms of the EXAFS oscillation **(Figure S10b)**. All three c-MOFs were found to register characteristic Cu-Cu scattering peaks (~2.2 Å), akin to Cu foils. The first peaks at ~1.44 Å for Cu_3_(HITP)_2_, Cu_3_(HHTP)_2_ and Cu_3_(THT)_2_ were attributed to Cu-X coordination. ^[^[[16]](#endnote-17)^]^


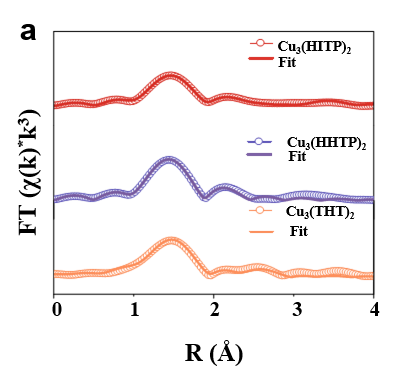


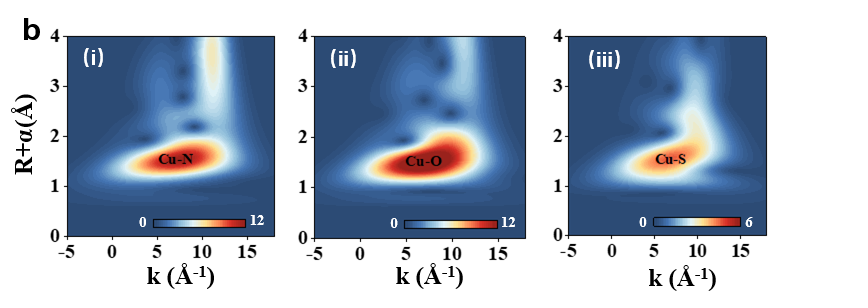


**Figure S11.** (a) Magnitude parts of R′ space EXAFS spectra and fits of the Cu_3_(HITP)_2_, Cu_3_(HHTP)_2_, Cu_3_(THT)_2_ and CuTPPCN_c_ sample. Inset: the illustration of the CuX_4_ (X = O, N, S) coordination motif. Corresponding fitting results are listed in Table S2. (b) Wavelet transform contour plots of EXAFS for Cu_3_(HITP)_2_ (i), Cu_3_(HHTP)_2_ (ii), and Cu_3_(THT)_2_ (iii).

The R-space fitting curves were found to be in agreement with the experimental spectra. As shown in the **Table S2** and **Figure S11,** coordination numbers for each of the Cu-X moieties was determined to be 4, confirming the presence of periodic Cu-X_4_ structures.^[^[[17]](#endnote-18)^]^

## Section 7: S12 Hirshfeld charge transfer of c-MOFs.


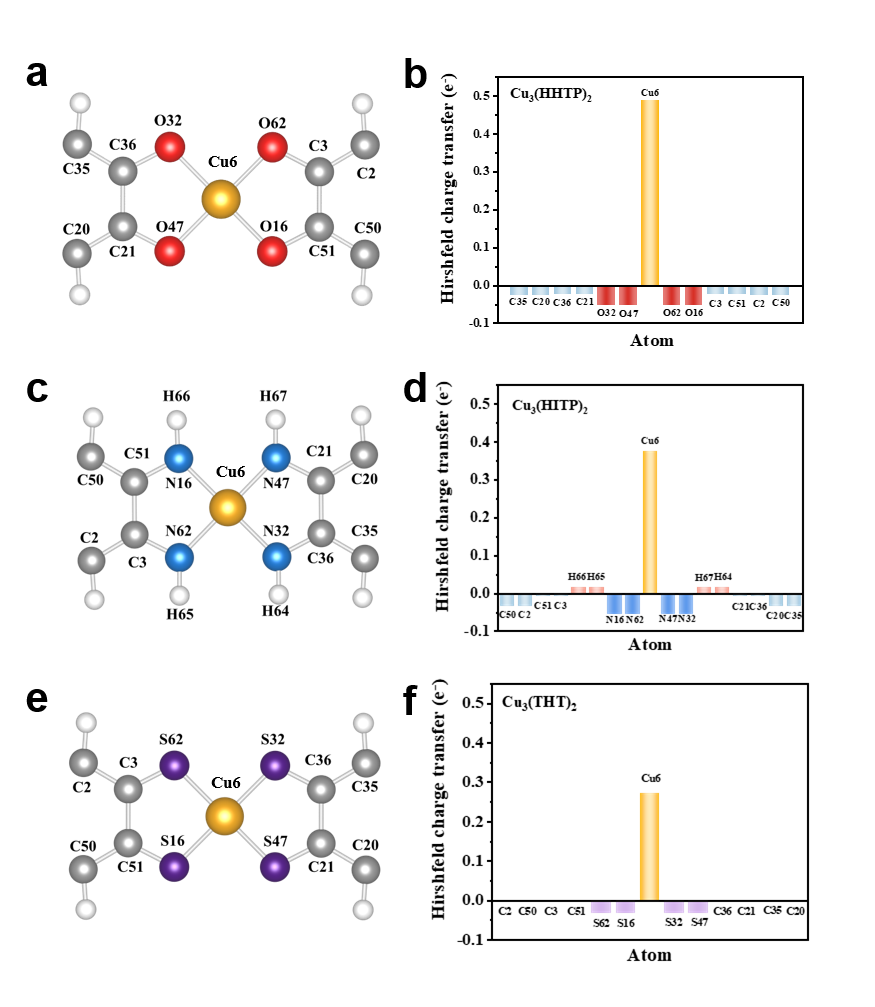


**Figure S12.** Numbering of atoms in (a) Cu_3_(HHTP)_2_, (c) Cu_3_(HITP)_2_ and (e) Cu_3_(THT)_2_. Partial atomic charge transfers in (b) Cu_3_(HHTP)_2_, (d) Cu_3_(HITP)_2_ and (f) Cu_3_(THT)_2_ are determined using the Hirshfeld charge scheme.

## Section 8: S13 Thermal stability of c-MOFs.


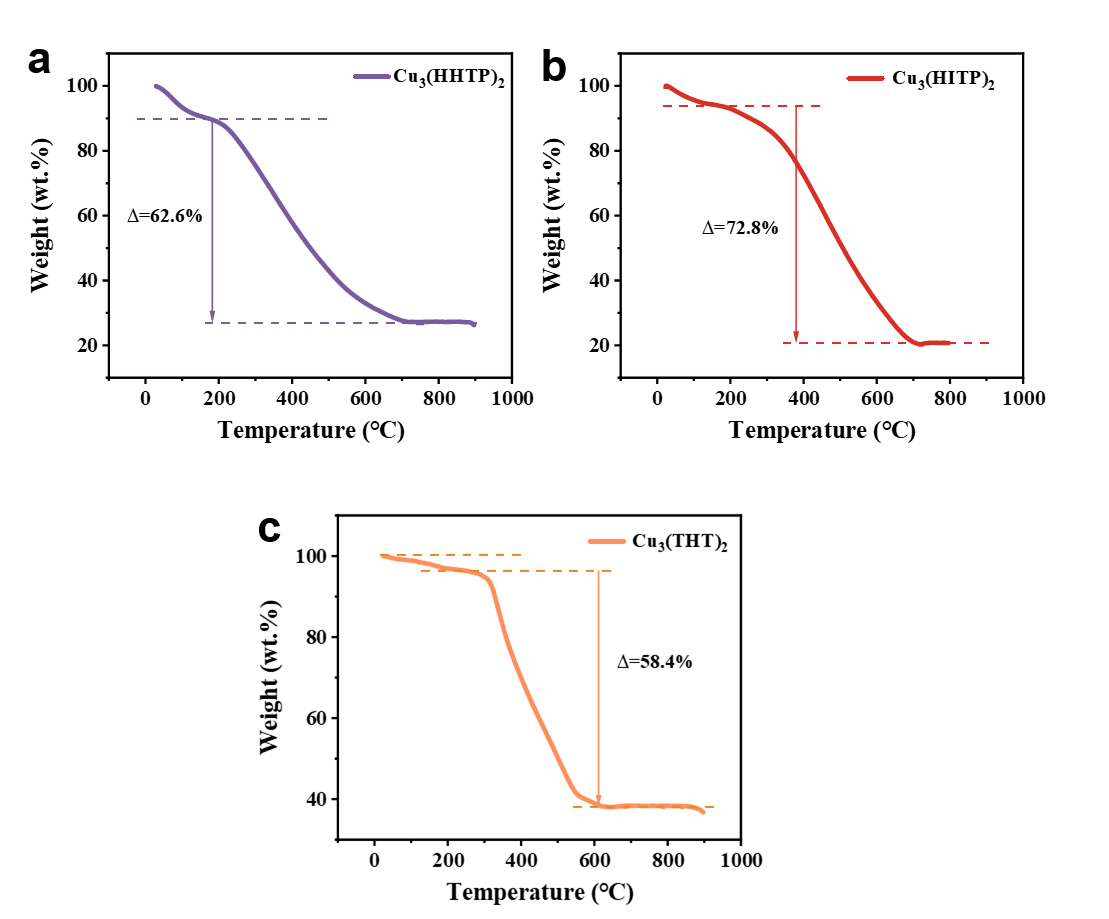


**Figure S13.** TGA traces under nitrogen flow, recorded for (a) Cu_3_(HHTP)_2_, (b) Cu_3_(HITP)_2_ and (c) Cu_3_(THT)_2_.

Thermogravimetric analysis (TGA) revealed that Cu_3_(THT)_2_ is more thermally stable than Cu_3_(HITP)_2_ and Cu_3_(HHTP)_2_, whose crystal structures are not collapsed until 300 °C. However, all three MOFs are stable below 200 °C, thus they still have the potential for application under high temperature conditions.

## Section 9: S14-S15 Electrically conductive properties of bulk of c-MOFs.


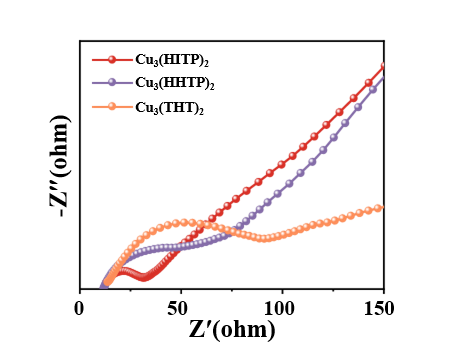


**Figure S14.** The Nyquist plots for Cu_3_(HHTP)_2_, Cu_3_(HITP)_2_, and Cu_3_(THT)_2_.

The Nyquist plot further supports that strong π-d conjugation significantly lowers the charge transfer resistance (R_ct_) in the three studied c-MOFs.


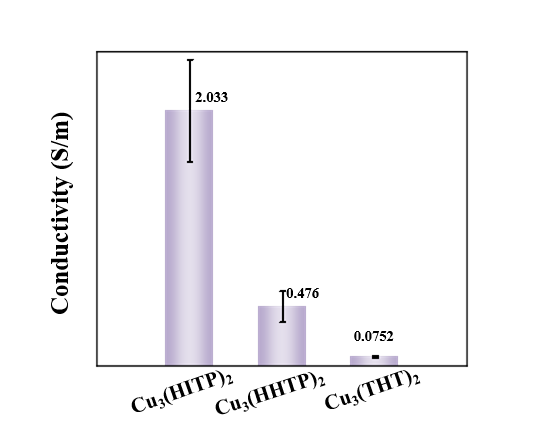


**Figure S15.** The conductivity of the Cu_3_(HITP)_2_, Cu_3_(HHTP)_2_, and Cu_3_(THT)_2_.

Four-probe conductivity test after pressing the sample powder into discs.

**Section 10: S16 Band structure of monolayer c-MOFs.**


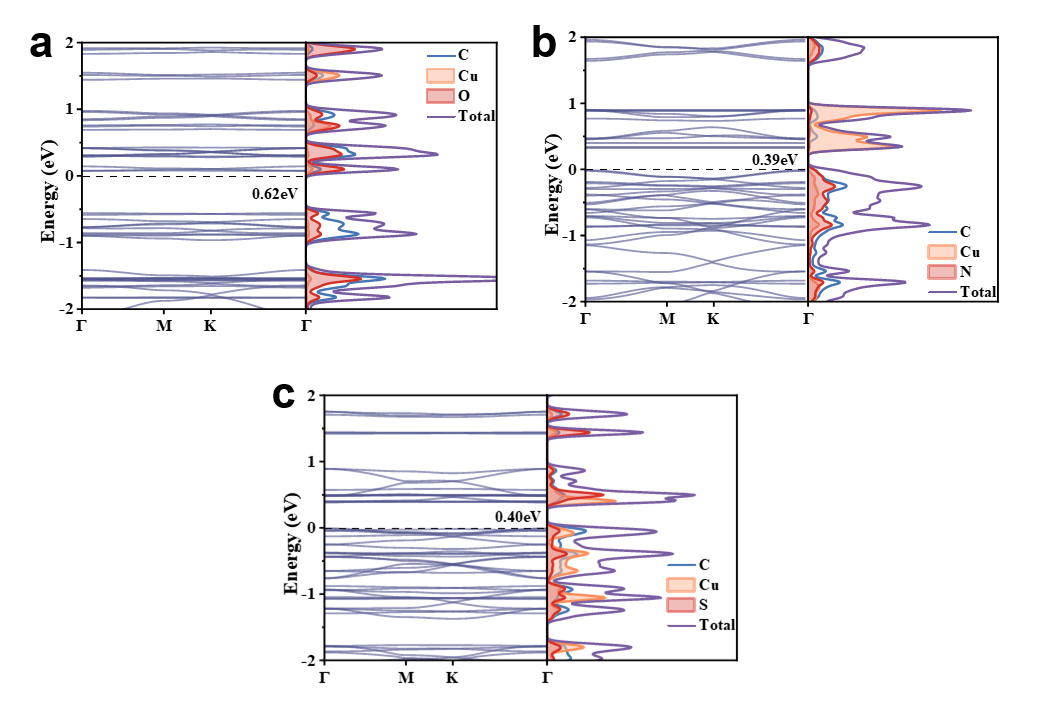


**Figure S16.** Electronic band structure and DOS of the simulated (a) Cu_3_(HHTP)_2_, (b) Cu_3_(HITP)_2_ and (c) Cu_3_(THT)_2_.

## Section 11: S17 Diffuse reflectance UV-vis-NIR spectra of c-MOFs.


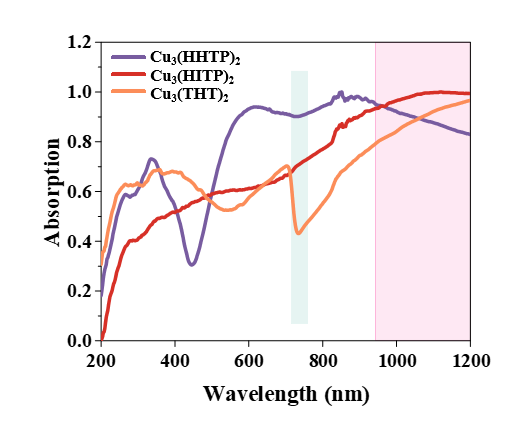


**Figure S17.** Diffuse reflectance UV-vis-NIR spectra of Cu_3_(HHTP)_2_, Cu_3_(HITP)_2_ and Cu_3_(THT)_2_; data, when normalized.

The broad peak in the spectrum centered at 730 nm is attributed to the inter-zone charge transfer (IVCT) between the Cu^+^ and Cu^2+^ centers, which is characteristic of mixed-valence compounds of class II in the Robin-Day classification.[^[[18]](#endnote-19)^] The absorption of Cu_3_(HITP)_2_ is significantly stronger in the near-infrared range (900-1200 nm), which indicates a very high degree of in-plane π-d electron delocalization in Cu_3_(HITP)_2_, suggesting stronger electronic coupling between the Cu and HITP ligands.^[^^[[19]](#endnote-20),^^[[20]](#endnote-21)]^

## Section 12: S18 EPR spectra of c-MOFs.


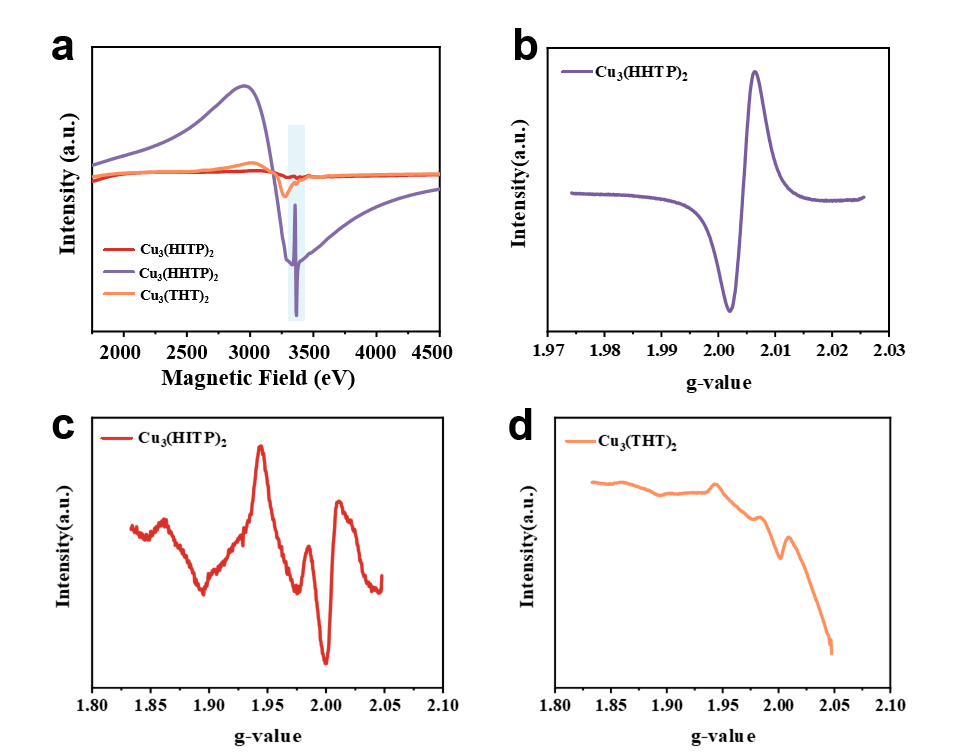


**Figure S18.** Electron paramagnetic resonance (EPR) spectra of Cu_3_(HHTP)_2_, Cu_3_(HITP)_2_ and Cu_3_(THT)_2_ at 77 K (a). Ligand-based radicals observed for c-MOFs (b-c) (g-factors: 2.005, 2.02, and 2.01, respectively.).


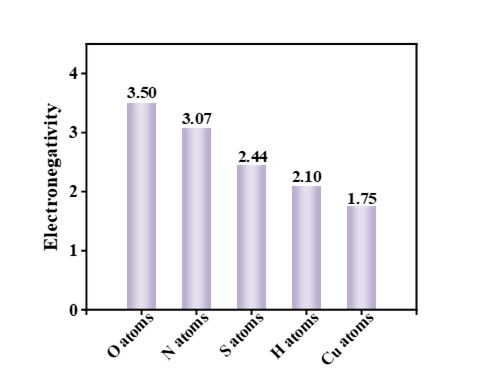


**Figure S19.** Comparison of electronegativity of O, N, S, H, and Cu. ^[^^[[21]](#endnote-22)]^

## Section 13: S20-S29 Electromagnetic wave absorption properties


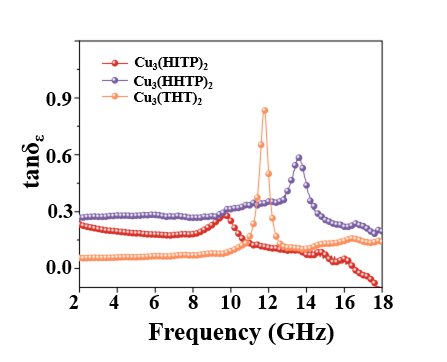


**Figure S20.** The dielectric loss tangent (tan δ_ε_) for Cu_3_(HHTP)_2_, Cu_3_(HITP)_2_, and Cu_3_(THT)_2_.


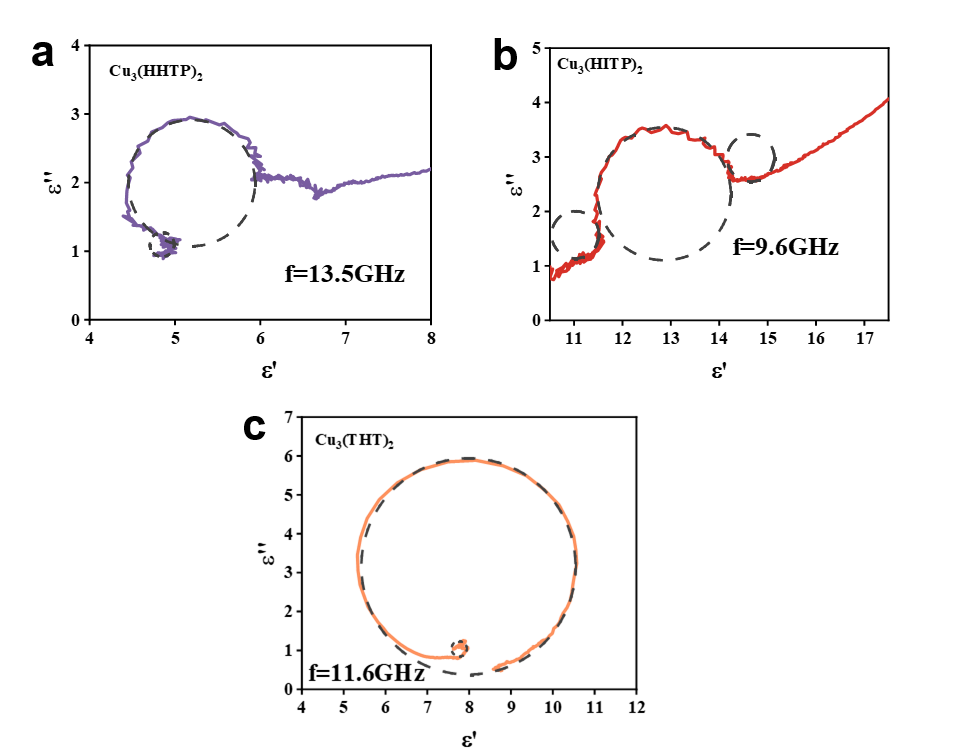


**Figure S21.** Cole-Cole semicircles of (a) Cu_3_(HHTP)_2_, (b) Cu_3_(HITP)_2_, and (c) Cu_3_(THT)_2_. The frequency of occurrence of prominent semicircles is labeled.

According to the Debye relaxation theory, the semicircular equation of the Cole-Cole circle is as follows: [^[[22]](#endnote-23)^]

$$\left[ \varepsilon^{'}-\frac{{(\varepsilon}_{s}+\varepsilon_{\infty})}{2} \right]^{2}+{\varepsilon^{''}}^{2}=\frac{{{(\varepsilon}_{s}-\varepsilon_{\infty})}^{2}}{4} (Equation S1)$$

Consequently, the plot of ε″/ε′ was noted as the Cole-Cole curve. The semicircle represents the polarization-relaxation process, and the upper right tail represents the conductive loss.


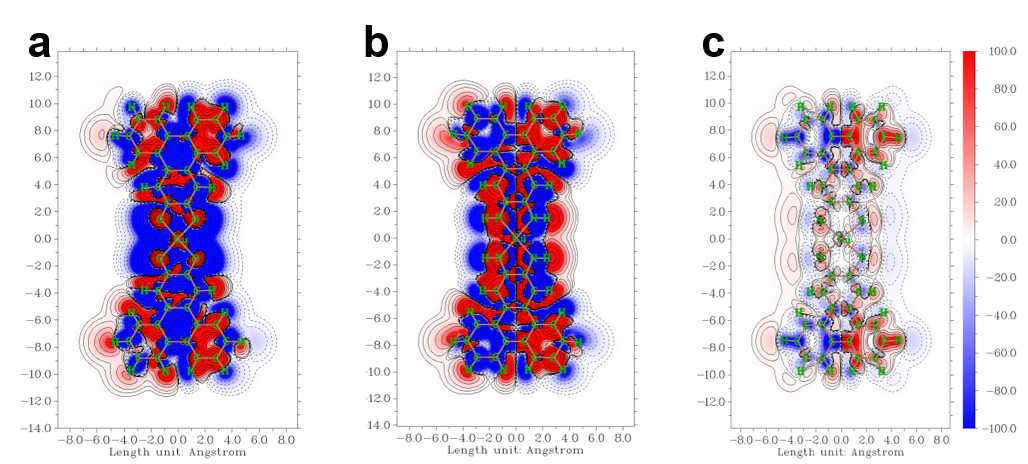


**Figure S22.** Hypolarizability density for (a) Cu_3_(HHTP)_2_, (b) Cu_3_(HITP)_2_ and (c) Cu_3_(THT)_2_.


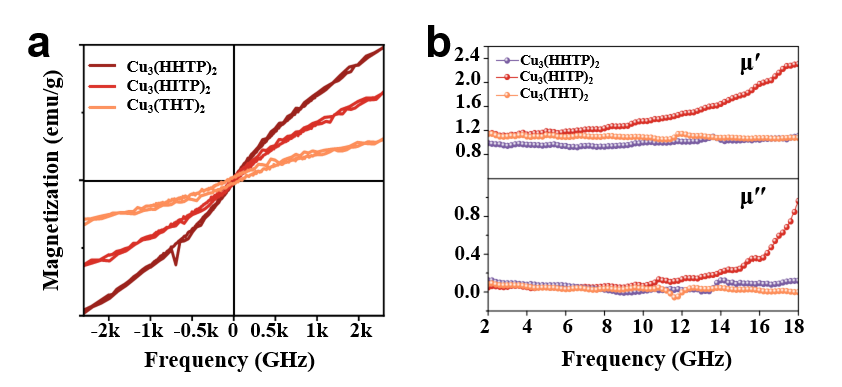


**Figure S23.** Magnetic properties. (a) Magnetization strength and of Cu_3_(HHTP)_2_, Cu_3_(HITP)_2_ and Cu_3_(THT)_2_. (b) The real part (μ′) and imaginary part (μ′′) Magnetic permeability of the Cu_3_(HHTP)_2_, Cu_3_(HITP)_2_, and Cu_3_(THT)_2_.


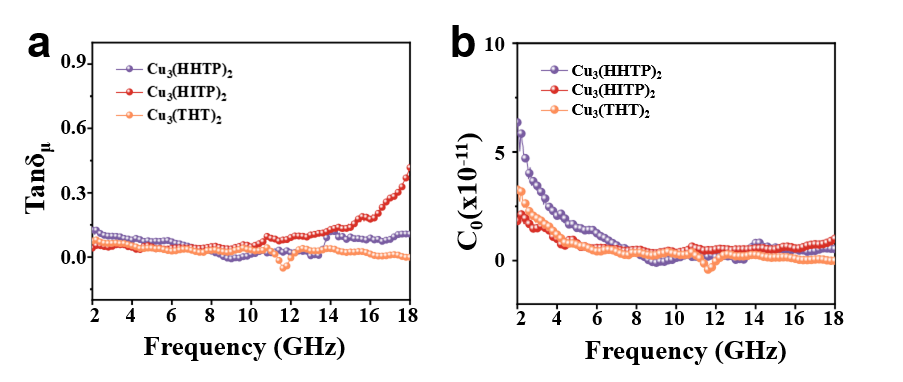


**Figure S24.** Magnetic loss mechanism analysis. (a) tanδ_µ_ of Cu_3_(HHTP)_2_, Cu_3_(HITP)_2_ and Cu_3_(THT)_2_. (b) C_0_ value of Cu_3_(HHTP)_2_, Cu_3_(HITP)_2_ and Cu_3_(THT)_2_.

 The eddy current loss ($C_{0}$) is evaluated using: [^[[23]](#endnote-24)^]

$$C_{0}=\frac{\mu^{''}}{\mu^{'2}f}=\frac{2\pi\sigma\mu_{0}d^{2}}{3} (Equation S2)$$

where $\mu_{0}$ denotes the vacuum permeability. $C_{0}$ is positively correlated with $d^{2}$and $\sigma$ . The$C_{0}$ value is almost stable in the range of 8–18 GHz in the $C_{0}$ -*f* curves (Figure S18), indicating a magnetic loss from the eddy current loss.

For the magnetic permeability, only Cu_3_(HITP)_2_ shows a significant increase in μ′ and μ′′ above 10 GHz **(Figure S23-S24)**. This phenomenon is mainly because the high charge transport property of Cu_3_(HITP)_2_ promotes the formation of annular induced currents at high-frequency electromagnetic fields, which increases the eddy current losses **(Equation S2).** However, the increase in induced currents may also lead to an increase in leakage currents, which results in a decrease in the dielectric constant. This behavior is consistent with the observed decrease in the dielectric constant of Cu_3_(HITP)_2_ at high frequencies.


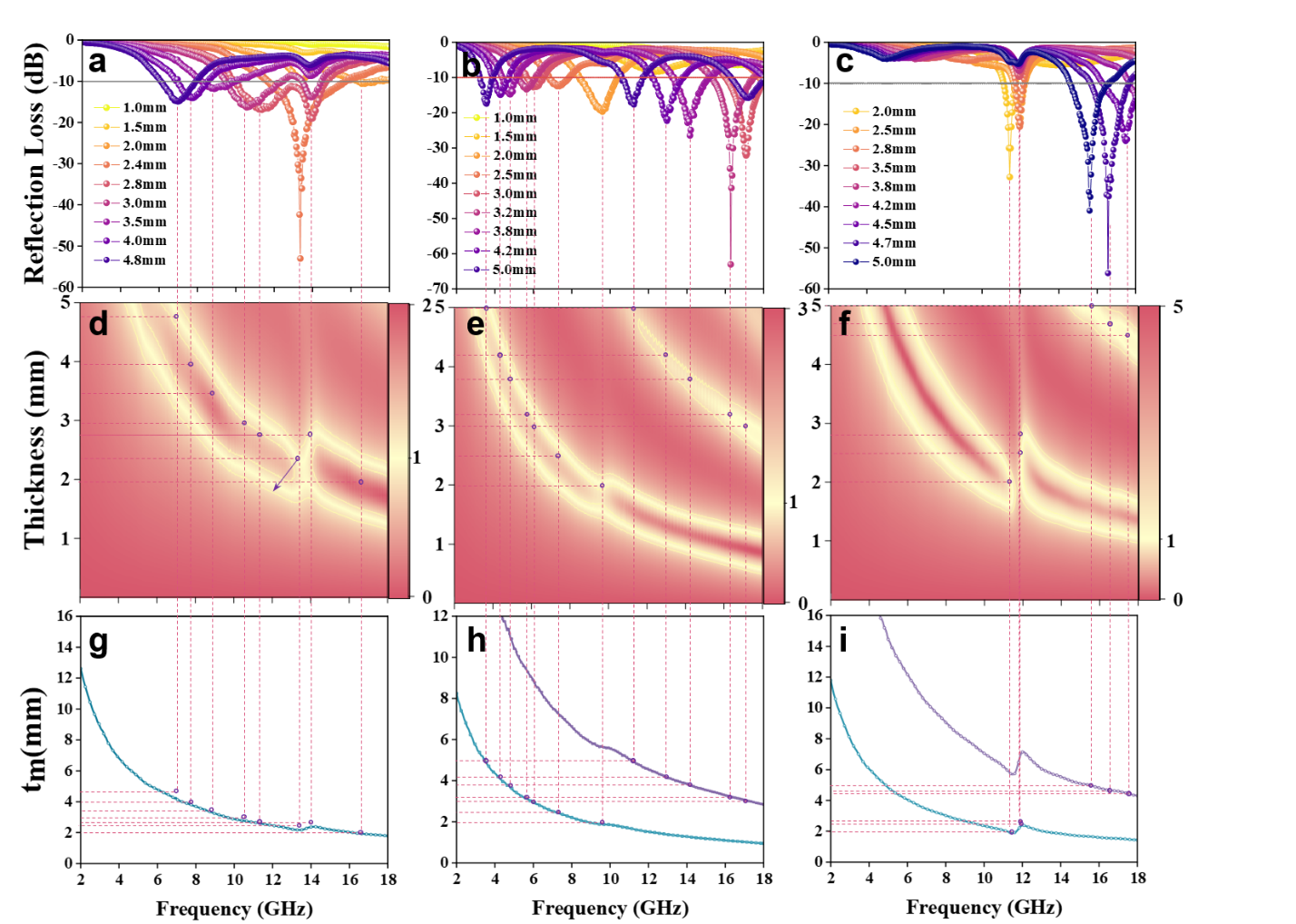


**Figure S25.** EMW absorption and impedance matching characteristic. (a-c) RL values at different thicknesses of Cu_3_(HHTP)_2_ (a), Cu_3_(HITP)_2_ (b) and Cu_3_(THT)_2_ (c). (d-f) 2D projections of the(Z′) and (Z″) values correlating the thickness and the frequencies of Cu_3_(HHTP)_2_ (d), Cu_3_(HITP)_2_ (e) and Cu_3_(THT)_2_ (f). (g-i) Theoretical matching thickness curves of Cu_3_(HHTP)_2_ (g), Cu_3_(HITP)_2_ (h) and Cu_3_(THT)_2_ (i).

Although Cu_3_(HITP)_2_ has considerable attenuation capability, the bandwidth is narrow. This is better explained by applying the interference cancellation theory proved by the 1/4λ theory. The formula is as follows: [^[[24]](#endnote-25)^]

$$t_{m}=\frac{n\lambda}{4}=\frac{nc}{4f_{m}\sqrt{\left| \mu_{r} \right|\left| \varepsilon_{r} \right|}} \left( n=1, 3, 5\ldots\ldots\right) (Equation S3)$$

$t_{m}$ and $f_{m}$ denote the matched thickness and the matched peak frequency, respectively; λ is the length of the electromagnetic wave; and c is the speed of light. When the absorber has a matching thickness that conforms to this equation, the incident and reflected waves differ by 180°, and due to the principle of wave interference, the two are completely phase cancelled at the absorber interface. This interference cancellation effect greatly enhances the attenuation of electromagnetic waves ^[^^[[25]](#endnote-26)]^. The results of the formula calculation are plotted in **Figure S25**, which shows that the$t_{m}$ exp of Cu_3_(HITP)_2_ is perfectly matched with tm siu, while the tm exp of Cu_3_(HHTP)_2_ deviates from tm siu to some extent. It shows that Cu_3_(HITP)_2_ relies more on interference cancellation, and when the matching thickness changes slightly, the perfect interference cancellation in Cu_3_(HITP)_2_ is damaged and affected more significantly than in Cu_3_(HHTP)_2_, resulting in a worse impedance match than Cu_3_(HHTP)_2_. Cu_3_(HITP)_2_ exhibits a pronounced dependence on destructive interference when satisfying the 1/4λ condition. The properties intensify the material's attenuation effects, but enhance its susceptibility to interference. The $t_{m}$ exp and $t_{m}$ siu of Cu_3_(THT)_2_ are also relatively matched, and the minimum reflection loss can reach -56.16 dB, but the bandwidth is only 2.24 GHz.


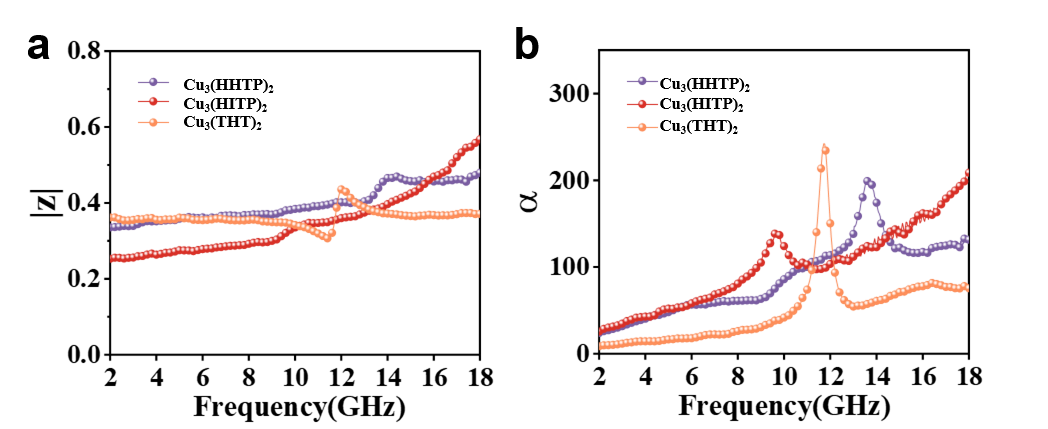


**Figure S26.** (a) Impedance matching and (b) attenuation constant of Cu_3_(HHTP)_2_, Cu_3_(HITP)_2_ and Cu_3_(THT)_2_.

The loss capacity was assessed by impedance matching (|Z|) and attenuation constant (α) as follows: [^[[26]](#endnote-27)^]

$$\left| Z \right|=\left| \frac{Z_{in}}{Z_{0}} \right| (Equation S4)$$

$$\alpha=\frac{\sqrt{2}\pi f}{c}\times\sqrt{\left( \mu^{''}\varepsilon^{''}-\mu^{'}\varepsilon^{'} \right)+\sqrt{\left( \mu^{''}\varepsilon^{''}-\mu^{'}\varepsilon^{'} \right)^{2}+\left( \mu^{''}\varepsilon^{'}+\mu^{'}\varepsilon^{''} \right)^{2}}} (Equation S5)$$

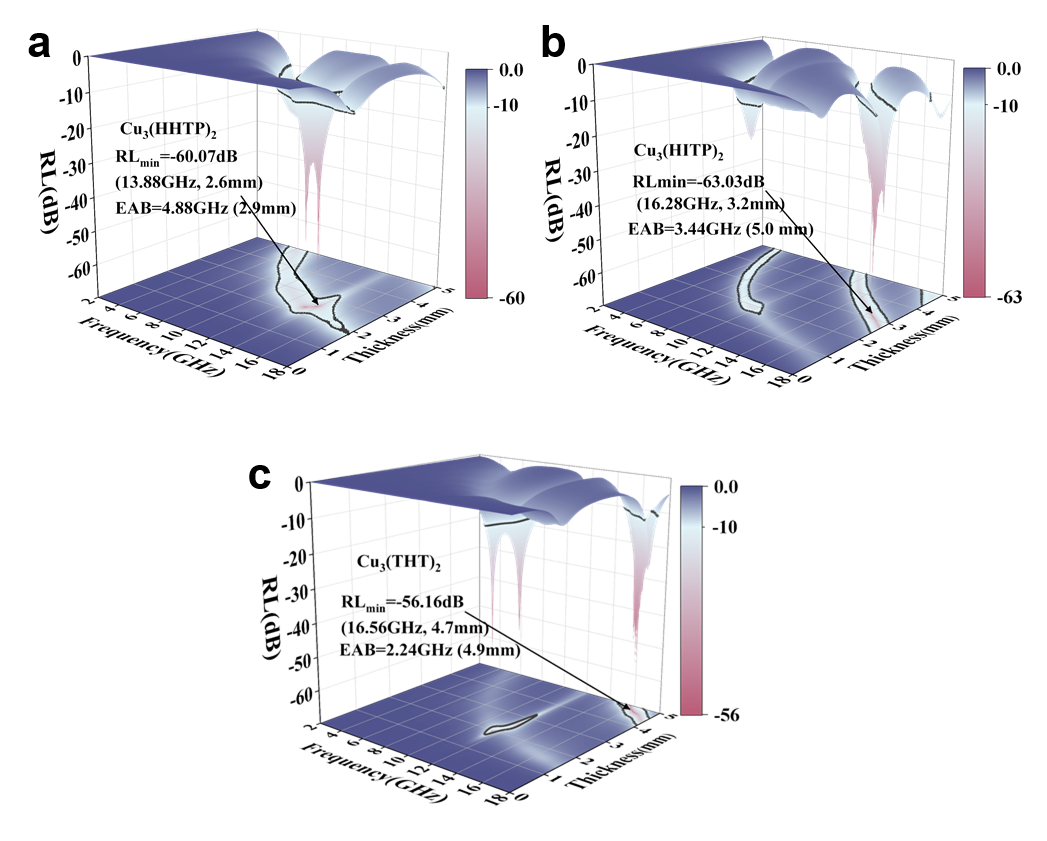


**Figure S27.** The 3D RL plots of (a) Cu_3_(HHTP)_2_, (b) Cu_3_(HITP)_2_ and (c) Cu_3_(THT)_2_ with 60% filling. 3D waterfall plot of thickness-frequency-reflection loss correspondence.

**
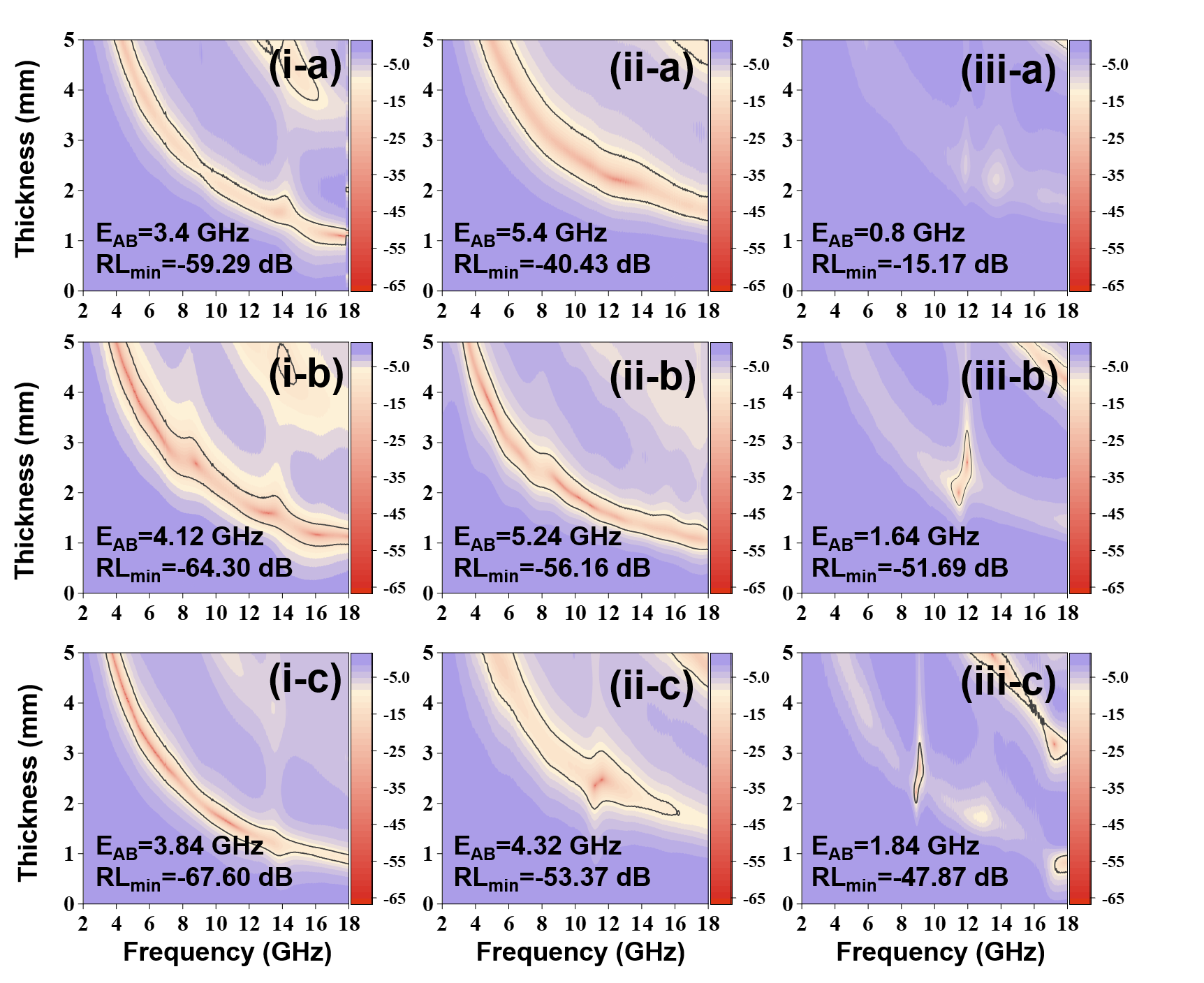
**

**Figure S28.** 3D contour projection maps of RL-frequency-thickness for Cu_3_(HITP)_2_ (i), Cu_3_(HHTP)_2_ (ii), and Cu_3_(THT)_2_ (iii) at filler ratios of 50 (a), 70 (b) and 80 (c) wt.%.


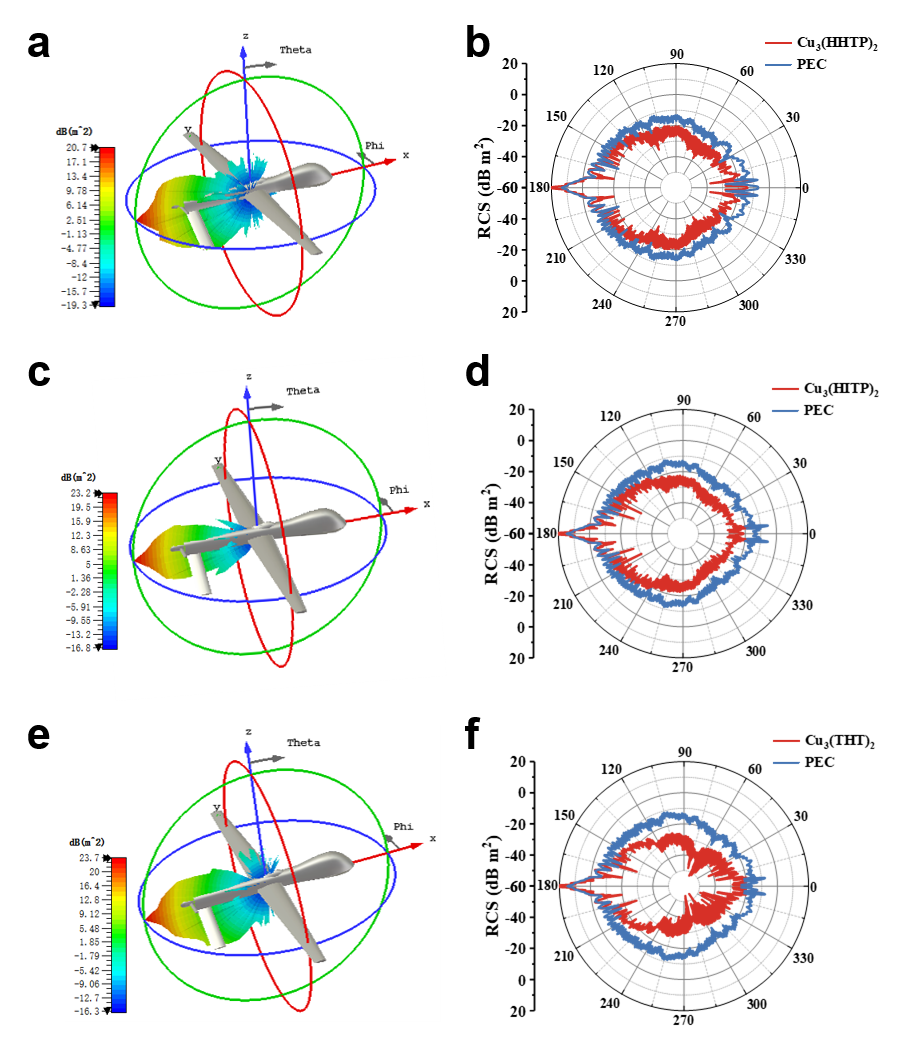


**Figure S29.** The Predator II model simulated radar transmission angles of 90° at the frequency of the strongest EMW absorption of (a, b) Cu_3_(HHTP)_2_ at 13.88 GHz, (c, d) Cu_3_(HITP)_2_ at 16.28 GHz and (e, f) Cu_3_(THT)_2_ at 16.56 GHz.


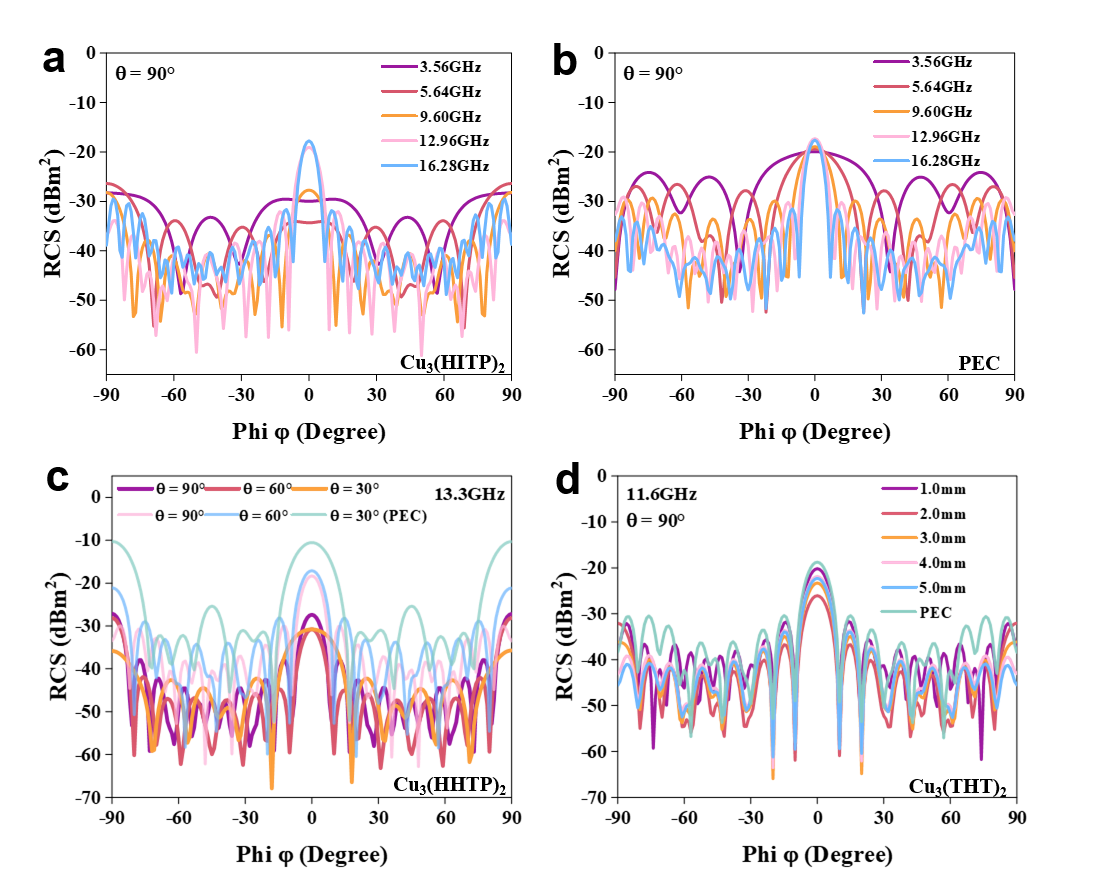


**Figure S30.** RCS values for metal backplane coated with (a) Cu_3_(HITP)_2_ at different frequencies, (b) PEC at frequencies corresponding to Cu_3_(HITP)_2_, (c) Cu_3_(HHTP)_2_ at 13.3 GHz at coating thicknesses of 2.4mm and (d) Cu_3_(THT)_2_ at 11.6 GHz at various coating thicknesses.

(a,b) For Cu_3_(HITP)_2_

The incident azimuth angles were restricted within the "-90° ≤ φ ≤ 90°, θ = 90° " condition at frequencies of 3.56 GHz, 5.64mm, 9.60 GHz and 16.28 GHz, with corresponding coating thicknesses of 5.0 mm, 3.4mm, 2.0 mm, and 3.2 mm.

For Cu_3_(HITP)_2_, frequencies of 3.56 GHz, 9.60 GHz, 12.96 GHz, and 16.28 GHz within the S, C, X, and Ku bands were selected, with corresponding coating thicknesses of 4.9 mm, 2.0 mm, 4.2 mm, and 3.2 mm. The RCS values for Cu_3_(HITP)_2_ remained below -10 dBm^2^ across most angular ranges.

(c) For Cu_3_(HHTP)_2_

The incident azimuth angles were restricted within the "-90° ≤ φ ≤ 90°, θ = 30°, 60°and 90° " condition at the frequency of 13.3GHz with a thickness of 2.4 mm.

The Cu_3_(HHTP)_2_ exhibited RCS values below -20 dBm^2^ at 13.3 GHz with a thickness of just 2.4 mm.

(d) For Cu_3_(THT)_2_

The incident azimuth angles were restricted within the "-90° ≤ φ ≤ 90°, θ = 30°, 60° and 90° " condition at the frequency of 11.6 GHz with thicknesses of 1.0 mm, 2.0 mm, 3.0 mm, 4.0 mm and 5.0 mm.

The Cu_3_(THT)_2_ demonstrated RCS values below -20 dBm^2^ at 11.6 GHz across a range of thicknesses, indicating its potential application as a frequency-specific absorber in antennas (Figure S30). These results suggest that by tuning the material’s coating thickness, effective absorption can be achieved across different frequency bands, highlighting the broader applicability of MOF materials in EMA field.

*
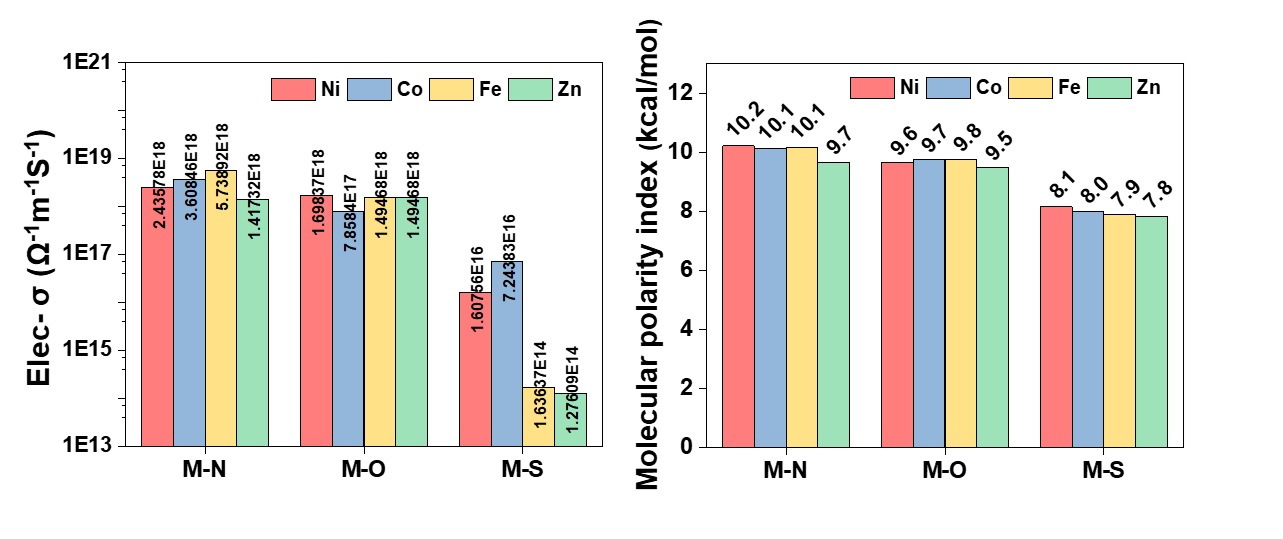
*

**Figure S31.** Theoretical calculations of electronic conductivity and molecular polarity index of MOFs with different metal centers.

# 4. Supplementary Tables

**Table S1.** Semi-quantitative XPS analysis of Cu_3_(HHTP)_2_, Cu_3_(HITP)_2_ and Cu_3_(THT)_2_ (integrated area of Cu^+^ as well as Cu^2+^ peaks)

|  | **Cu^2+^** | **Cu^+^** | **Cu^2+^/Cu^+^ ratio** |
| --- | --- | --- | --- |
| Cu_3_(HHTP)_2_ | 0.627 | 0.150 | 4.18 |
| Cu_3_(HITP)_2_ | 0.448 | 0.423 | 1.06 |
| Cu_3_(THT)_2_ | 0.488 | 0.356 | 1.37 |

**Table S2.** Parameters of the Cu K-edge EXAFS fitting results for Cu_3_(HHTP)_2_, Cu_3_(HITP)_2_ and Cu_3_(THT)_2_.

|  | **Scattering pair** | **coordination number** | **R(Å)** | **σ^2^**  **(10^-3^Å^2^)** | **ΔE_0_** | **R-factor** |
| --- | --- | --- | --- | --- | --- | --- |
| Cu_3_(HHTP)_2_ | Cu-O | 3.92 | 1.93 | 5.3 | -7.42 | 0.0114 |
| Cu_3_(HITP)_2_ | Cu-N | 3.8 | 1.98 | 5.3 | -5.51 | 0.00643 |
| Cu_3_(THT)_2_ | Cu-S | 3.8 | 1.77 | 15.26 | 10.15 | 0.0205 |

Note:

R, distance between absorber and backscatter atoms (Bond length between the center atom and the surrounding ligand atoms);

σ^2^, Debye–Waller factor to describe the variance due to disorder (An index for measuring thermal and static disorder in absorber scattering distances);

ΔE_0_, the inner potential correction (Difference between the zero kinetic energy value of the specimen and the zero kinetic energy value of the theoretical model);

R-factor is used to evaluate the quality of the fitting and the smaller value means more satisfied fitting.

**Table S3.** Sum of quantitative contributions to polarizability from individual atoms in Cu_3_(HHTP)_2_, Cu_3_(HITP)_2_ and Cu_3_(THT)_2_.

|  | **Cu_3_(HHTP)_2_** | **Cu_3_(HITP)_2_** | **Cu_3_(THT)_2_** |
| --- | --- | --- | --- |
| Sum Value | 30221.65944277 | 30872.00423022 | 29869.28839318 |

Obtained by integrating the hyperpolarizability density in atomic space, it can be used to analyze the quantitative contribution to the polarizability.

**Table S4.** The comparison between typical Cu-based materials and other metal-conductive MOFs

| **Sample** | **RLmin (dB)** | **EAB (GHz)** | **Loading (%)** | **Ref.** |
| --- | --- | --- | --- | --- |
| CuHT | −50.9 | 4.2 | 50 | [^[[27]](#endnote-28)^] |
| Zn_3_(HHTP)_2_ | -30.5 | 2.59 | 60 | [^[[28]](#endnote-29)^] |
| Co-HHTP | −38.06 | 4.2 | 50 | [2] |
| MoS_2_ | -45.5 | 3.89 | 50 | [^[[29]](#endnote-30)^] |
| Cu-MOF-74 | −15.1 | 6.2 | 40 | [^[[30]](#endnote-31)^] |
| C/Cu/Cu_2_O/Cu_2_−xS | -33.5 | 7.6 | 40 | [^[[31]](#endnote-32)^] |
| Cu_9_S_5_/NC@Co_3_S_4_/NC | -41.6 | 4.08 | 52 | [^[[32]](#endnote-33)^] |
| Cu/C MM-700 | -52.44 | 6.8 | 40 | [^[[33]](#endnote-34)^] |
| Cu9S5/C | -62.3 | 4.7 | 45 | [^[[34]](#endnote-35)^] |
| CuNi-3/ACET | –40.6 | 5.87 | 40 | [^[[35]](#endnote-36)^] |
| Cu/CuO/C | -39 | 5.5 | 50 | [^[[36]](#endnote-37)^] |
| CuS | -31.5 | 3.6 | 30 | [^[[37]](#endnote-38)^] |

# 3. Reference

1. [] X. Song, X. Wang, Y. Li, C. Zheng, B. Zhang, C. Di, F. Li, C. Jin, W. Mi, L. Chen, W. Hu, Angew. Chem. Int. Ed., 2020, 59, 1118–1123. [↑](#endnote-ref-2)
2. [] N.-Y. Huang, H. He, S. Liu, H.-L. Zhu, Y.-J. Li, J. Xu, J.-R. Huang, X. Wang, P.-Q. Liao, X.-M. Chen, J. Am. Chem. Soc. 2021, 143, 17424–17430. [↑](#endnote-ref-3)
3. [] [1] K. W. Nam, S. S. Park, R. dos Reis, V. P. Dravid, H. Kim, C. A. Mirkin, J. F. Stoddart, Nat Commun 2019, 10, 4948. [↑](#endnote-ref-4)
4. [] N. Contreras-Pereda, S. Pané, J. Puigmartí-Luis, D. Ruiz-Molina, Coordination Chemistry Reviews 2022, 460, 214459. [↑](#endnote-ref-5)
5. [] R. Dong, P. Han, H. Arora, M. Ballabio, M. Karakus, Z. Zhang, C. Shekhar, P. Adler, P. S. Petkov, A. Erbe, S. C. B. Mannsfeld, C. Felser, T. Heine, M. Bonn, X. Feng, E. Cánovas, Nature Mater 2018, 17, 1027–1032. [↑](#endnote-ref-6)
6. [] J.-D. Yi, R. Xie, Z.-L. Xie, G.-L. Chai, T.-F. Liu, R.-P. Chen, Y.-B. Huang, R. Cao, Angew. Chem. Int. Ed., 2020, 59, 23641–23648. [↑](#endnote-ref-7)
7. [] A. J. Clough, J. W. Yoo, M. H. Mecklenburg, S. C. Marinescu, J. Am. Chem. Soc. 2015, 137, 118–121. [↑](#endnote-ref-8)
8. [] K. Fan, C. Zhang, Y. Chen, Y. Wu, C. Wang, Chem 2021, 7, 1224–1243. [↑](#endnote-ref-9)
9. [] A. Nazir, H. T. T. Le, A. Kasbe, C.-J. Park, Chemical Engineering Journal 2021, 405, 126963. [↑](#endnote-ref-10)
10. [] L. Mendecki, M. Ko, X. Zhang, Z. Meng, K. A. Mirica, J. Am. Chem. Soc. 2017, 139, 17229–17232. [↑](#endnote-ref-11)
11. [] C. Meng, P. Hu, H. Chen, Y. Cai, H. Zhou, Z. Jiang, X. Zhu, Z. Liu, C. Wang, A. Yuan, Nanoscale 2021, 13, 7751–7760. [↑](#endnote-ref-12)
12. [] P. Zhang, Y. Li, Y. Zhang, R. Hou, X. Zhang, C. Xue, S. Wang, B. Zhu, N. Li, G. Shao, Small Methods 2020, 4, 2000214. [↑](#endnote-ref-13)
13. [] W. Xiong, D. Si, J. Yi, Y. Huang, H. Li, R. Cao, Applied Catalysis B: Environmental 2022, 314, 121498. [↑](#endnote-ref-14)
14. [] W. Xiong, D. Si, J. Yi, Y. Huang, H. Li, R. Cao, Applied Catalysis B: Environmental 2022, 314, 121498. [↑](#endnote-ref-15)
15. [] X. Sun, Y. Li, H. Su, X. Zhang, Y. Xu, W. Zhou, M. Liu, W. Cheng, Q. Liu, Applied Catalysis B: Environmental 2022, 317, 121706. [↑](#endnote-ref-16)
16. [] P. Yu, X. Lv, Q. Wang, H. Huang, W. Weng, C. Peng, L. Zhang, G. Zheng, Small 2023, 19, 2205730. [↑](#endnote-ref-17)
17. [] H. Funke, A. C. Scheinost, M. Chukalina, Phys. Rev. B 2005, 71, 094110. [↑](#endnote-ref-18)
18. [] M. B. Robin, P. Day, in Advances in Inorganic Chemistry and Radiochemistry (Eds.: H.J. Emeléus, A.G. Sharpe), Academic Press, 1968, pp. 247–422. [↑](#endnote-ref-19)
19. [] J. Wang, T. Chen, M. Jeon, J. J. Oppenheim, B. Tan, J. Kim, M. Dincă, J. Am. Chem. Soc. 2024, DOI 10.1021/jacs.4c06935. [↑](#endnote-ref-20)
20. [] P. Apostol, S. M. Gali, A. Su, D. Tie, Y. Zhang, S. Pal, X. Lin, V. R. Bakuru, D. Rambabu, D. Beljonne, M. Dincă, A. Vlad, J. Am. Chem. Soc. 2023, 145, 24669–24677. [↑](#endnote-ref-21)
21. [] E. J. Jr. Little, M. M. Jones, J. Chem. Educ. 1960, 37, 231. [↑](#endnote-ref-22)
22. [] Z. Shan, S. Cheng, F. Wu, X. Pan, W. Li, W. Dong, A. Xie, G. Zhang, Chemical Engineering Journal 2022, 446, 137409. [↑](#endnote-ref-23)
23. [] B. Dai, Y. Ma, F. Dong, J. Yu, M. Ma, H. K. Thabet, S. M. El-Bahy, M. M. Ibrahim, M. Huang, I. Seok, G. Roymahapatra, N. Naik, B. B. Xu, J. Ding, T. Li, Adv Compos Hybrid Mater 2022, 5, 704–754. [↑](#endnote-ref-24)
24. [] X. Zhang, X.-L. Tian, Y. Qin, J. Qiao, F. Pan, N. Wu, C. Wang, S. Zhao, W. Liu, J. Cui, Z. Qian, M. Zhao, J. Liu, Z. Zeng, ACS Nano 2023, 17, 12510–12518. [↑](#endnote-ref-25)
25. [] J. Ruan, Z. Chang, H. Rong, T. S. Alomar, D. Zhu, N. AlMasoud, Y. Liao, R. Zhao, X. Zhao, Y. Li, B. B. Xu, Z. Guo, Z. M. El-Bahy, H. Li, X. Zhang, S. Ge, Carbon 2023, 213, 118208. [↑](#endnote-ref-26)
26. [] S. Cheng, X. Pan, A. Xie, J. Shi, Q. Qiu, C. Zhang, W. Dong, X. Qi, Chemical Engineering Journal 2021, 417, 127980. [↑](#endnote-ref-27)
27. [] P. Miao, T. Zhang, T. Wang, J. Chen, T. Gao, Y. Wang, J. Kong, K. Chen, Chin. J. Chem. 2022, 40, 467–474. [↑](#endnote-ref-28)
28. [] A. Dong, Z. Mu, X. Meng, S. Li, J. Li, L. Dai, J. Lv, P. Li, B. Wang, Chemical Engineering Journal 2022, 444, 136574. [↑](#endnote-ref-29)
29. [] M. Ning, P. Jiang, W. Ding, X. Zhu, G. Tan, Q. Man, J. Li, R.-W. Li, Advanced Functional Materials 2021, 31, 2011229. [↑](#endnote-ref-30)
30. [] W. Li, J. Chen, P. Gao, Journal of Colloid and Interface Science 2022, 606, 719–727. [↑](#endnote-ref-31)
31. [] Z. Zhao, K. Kou, L. Zhang, H. Wu, Carbon 2022, 186, 323–332. [↑](#endnote-ref-32)
32. [] H. Zhu, J. Liang, X. Jiao, R. Fu, Q. Jiao, C. Feng, H. Li, Y. Zhang, Y. Zhao, Ceramics International 2023, 49, 9534–9542. [↑](#endnote-ref-33)
33. [] Y. Jiao, S. Cheng, F. Wu, X. Pan, A. Xie, X. Zhu, W. Dong, Composites Part B: Engineering 2021, 211, 108643. [↑](#endnote-ref-34)
34. [] D. Xu, Y. Yang, K. Le, G. Wang, A. Ouyang, B. Li, W. Liu, L. Wu, Z. Wang, J. Liu, F. Wang, Chemical Engineering Journal 2021, 417, 129350. [↑](#endnote-ref-35)
35. [] J. Cheng, H. Zhang, H. Wang, Z. Huang, H. Raza, C. Hou, G. Zheng, D. Zhang, Q. Zheng, R. Che, Advanced Functional Materials 2022, 32, 2201129. [↑](#endnote-ref-36)
36. [] Y. Guo, Q. Chang, Z. Shi, J. Xie, J. Yun, L. Zhang, H. Wu, Journal of Colloid and Interface Science 2023, 639, 444–453. [↑](#endnote-ref-37)
37. [] B. Zhao, G. Shao, B. Fan, W. Zhao, Y. Xie, R. Zhang, J. Mater. Chem. A 2015, 3, 10345–10352. [↑](#endnote-ref-38)
